# Anoctamin 1 controls bone resorption by coupling Cl^−^ channel activation with RANKL-RANK signaling transduction

**DOI:** 10.1038/s41467-022-30625-9

**Published:** 2022-05-24

**Authors:** Weijia Sun, Shuai Guo, Yuheng Li, JianWei Li, Caizhi Liu, Yafei Chen, Xuzhao Wang, Yingjun Tan, Hua Tian, Cheng Wang, Ruikai Du, Guohui Zhong, Sai Shi, Biao Ma, Chang Qu, Jingxuan Fu, Xiaoyan Jin, Dingsheng Zhao, Yong Zhan, Shukuan Ling, Hailong An, Yingxian Li

**Affiliations:** 1 State Key Laboratory of Space Medicine Fundamentals and Application, China Astronaut Research and Training Center, 100094 Beijing, China; 2 Key Laboratory of Molecular Biophysics, Hebei Province, Institute of Biophysics, School of Health Sciences and Biomedical Engineering, Hebei University of Technology, 300401 Tianjin, China; 3 School of Life Science, Hebei University, 071002 Baoding, Hebei China; 4 The Key Laboratory of Aerospace Medicine, Ministry of Education, The Fourth Military Medical University, Xi’an, Shaanxi China; 5 Department of Orthopedics /Engineering Research Center of Bone and Joint Precision Medicine /Beijing Key Laboratory of Spinal Disease Research, Peking University the Third Hospital, 100191 China Beijing,

**Keywords:** Mechanisms of disease, Molecular biology, Calcium signalling

## Abstract

Osteoclast over-activation leads to bone loss and chloride homeostasis is fundamental importance for osteoclast function. The calcium-activated chloride channel Anoctamin 1 (also known as TMEM16A) is an important chloride channel involved in many physiological processes. However, its role in osteoclast remains unresolved. Here, we identified the existence of Anoctamin 1 in osteoclast and show that its expression positively correlates with osteoclast activity. Osteoclast-specific Anoctamin 1 knockout mice exhibit increased bone mass and decreased bone resorption. Mechanistically, Anoctamin 1 deletion increases intracellular Cl^−^ concentration, decreases H^+^ secretion and reduces bone resorption. Notably, Anoctamin 1 physically interacts with RANK and this interaction is dependent upon Anoctamin 1 channel activity, jointly promoting RANKL-induced downstream signaling pathways. Anoctamin 1 protein levels are substantially increased in osteoporosis patients and this closely correlates with osteoclast activity. Finally, Anoctamin 1 deletion significantly alleviates ovariectomy induced osteoporosis. These results collectively establish Anoctamin 1 as an essential regulator in osteoclast function and suggest a potential therapeutic target for osteoporosis.

## Abstract (web summary)

Osteoclast over-activation leads to bone loss and chloride homeostasis is important for osteoclast function. Here, the authors show that Anoctamin 1 controls bone resorption by coupling Cl^−^ channel activation with RANKL-RANK signaling transduction.

## Introduction

Osteoclasts are multinucleated giant cells that originate from the monocyte/macrophage hematopoietic lineage, which are the cells responsible for bone resorption, ensuring development, and continuous remodeling of the skeleton. Reduced osteoclast activity causes osteopetrosis, whereas enhanced activity leads to osteoporosis^1^. Ca^2+^ serves as a critical intracellular second messenger and Ca^2+^ signaling is the main mechanism that mediates osteoclast differentiation. The Ca^2+^/calmodulin-dependent protein kinases IV-cyclic AMP-responsive element-binding protein-nuclear factor of activated T cells c1 (CaMKIV-CREB-NFATc1) pathway plays an essential role in osteoclast differentiation and function.

Osteoclasts are equipped with an elaborate set of membrane transport proteins (pumps, transporters, and channels) that serve as molecular gatekeepers to regulate its function. Cl^−^ is the most abundant free anion in animal cells, and Cl^−^ channels have fundamental roles in the function of osteoclasts^2^. Two Cl^−^ transporters are known to exist in the ruffled border of osteoclasts. One of these transporters is chloride voltage-gated channel 7 (ClC-7), a lysosomal CLC family Cl^−^/H^+^ antiporter, which has been identified as the Cl^−^ transporter involved in the maintenance of electroneutrality in the resorption lacunae. Another transporter is the chloride intracellular channel 5 (CLIC5) channel, which is usually expressed intracellularly. There is no human bone disease related to CLIC5 dysfunction. To date, little is known about changes in Cl^−^ currents and their regulation on osteoclast development and bone resorption.

Ca^2+^-activated Cl^−^ channels (CaCCs) are plasma membrane proteins that are widely distributed in various tissues, indicating their diverse physiological functions. One of the best-known functions of CaCCs in mammals is Cl^−^ secretion in secretory epithelia. In 2008, three independent research teams identified Anoctamin 1 (Ano1), also known as TMEM16A and Anoctamin 2 (Ano2) as components of CaCCs, which are activated by an increase in the [Ca^2+^]_i_^3–5^. Ano1 is a homodimer formed by subunits that each consist of ten membrane-spanning ɑ-helices, and is involved in many physiological processes, such as fluid secretion in many secretory epithelia^6^, smooth muscle contraction^7^, nociception^8,9^, the formation of most tumors^10–13^, and cell proliferation^14,15^. However, its expression and role in osteoclasts remain unelucidated.

In this study, we report that Ano1 levels are positively correlated with osteoclast activity. In mice, osteoclast-specific *Ano1* knockout induces increased bone density and reduced bone resorption, and can alleviate ovariectomy (OVX)-induced bone loss. Ano1 is required for Cl^−^ efflux, H^+^ secretion, and osteoclast function, and can also interact with RANK and promote the activation of the RANKL–RANK pathway, which is dependent on CaCC activity. These results demonstrate that Ano1 in osteoclasts plays a crucial role in bone homeostasis by coupling Cl^−^ channel activity and RANK signaling, and that Ano1 is a promising therapeutic target for osteoporosis.

## Results

### Ano1 is the pivotal Cl^−^ channel for osteoclast differentiation

**Fig. 1:**
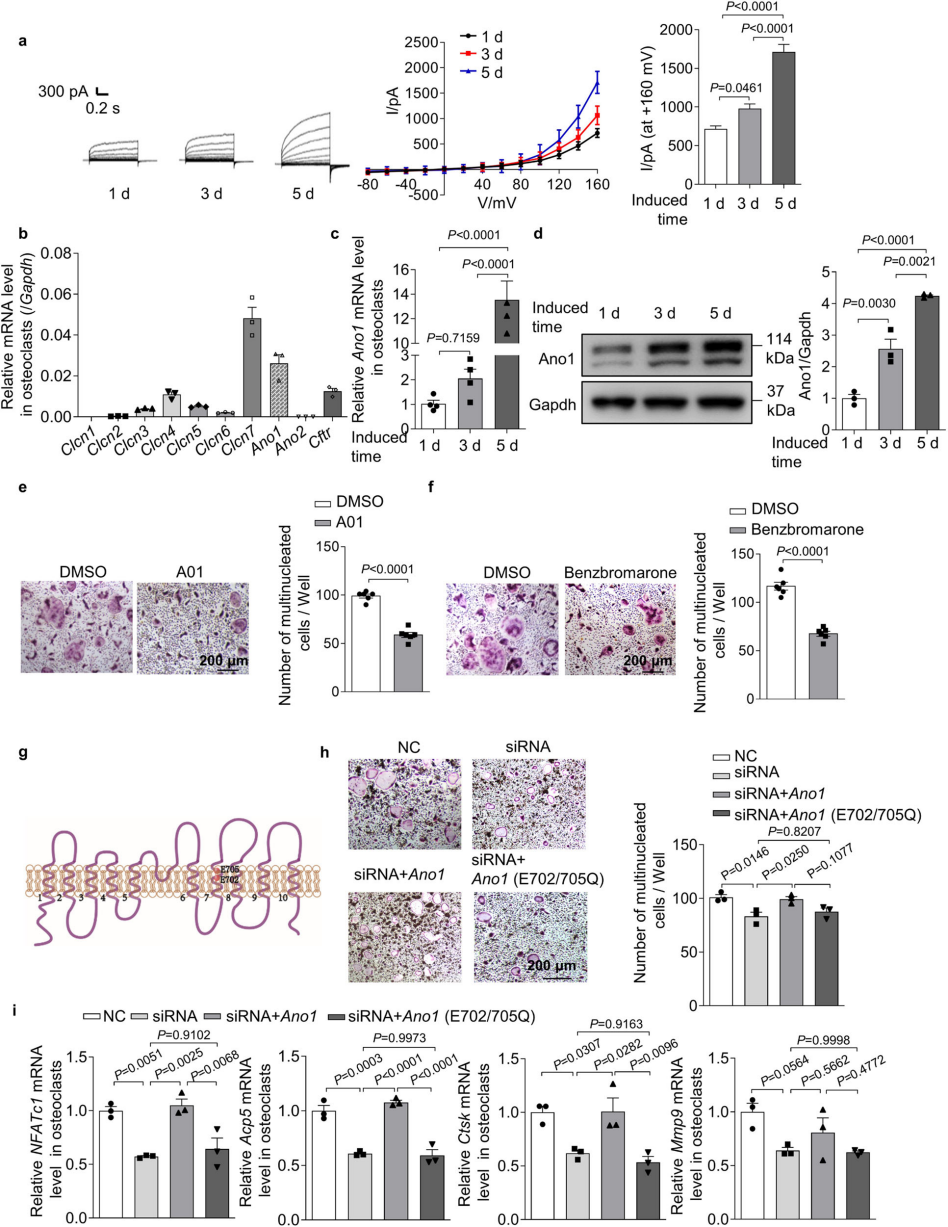
The expression and function of Ano1 in osteoclast. **a** Representative chloride currents recorded from voltage ramps from −80 to +160 mV in whole-cell patch-clamp during osteoclast differentiation. **b** QRT-PCR analysis of *Clcn1*, *Clcn2*, *Clcn3*, *Clcn4*, *Clcn5*, *Clcn6*, *Clcn7*, *Ano1*, *Ano2*, and *Cftr* mRNA levels after bone marrow monocytes (BMMs) were induced by RANKL for 5 days. **c**, **d** QRT-PCR analysis of *Ano1* mRNA level (**c**) and western blot analysis of Ano1 protein level (**d**) during osteoclast differentiation. **e** Representative images of TRAP staining in osteoclasts after treatment with 20 μM CaCC_inh_-A01 (A01) or its control (DMSO) for 5 days (left). Scale bar, 200 μm. Quantification of the number of multinucleated cells per well (right) (*n* = 6). **f** Representative images of TRAP staining in osteoclasts after treatment with 10 μM Benzbromarone or its control (DMSO) for 5 days (left). Scale bar, 200 μm. Quantification of the number of multinucleated cells per well (right) (*n* = 6). **g** Schematic representation of the topology of Ano1 mutant (E702/705Q). **h** Representative images of TRAP staining in osteoclasts knockdown with *Ano1* siRNA, rescued with *Ano1* or its mutant *Ano1* (E702/705Q) (left). Scale bar, 200 μm. Quantification of the number of multinucleated cells per well (right). **i** QRT-PCR analysis of *NFATc1*, *Acp5*, *Ctsk*, and *Mmp9* mRNA levels in osteoclasts knockdown with *Ano1* siRNA, rescued with *Ano1* or its mutant *Ano1* (E702/705Q). All data are the mean ± s.e.m. from three independent experiments. Two-tailed unpaired Student’s *t*-test was used for statistical evaluations of two group comparisons. Statistical analysis with more than two groups was performed with one-way analysis of variance (ANOVA) with Tukey’s multiple comparisons test to determine group differences. Source data are provided as a Source Data file.

We evaluated the change of Cl^−^ current during osteoclast differentiation. We found that there was a time-dependent increase in the outward-rectifying Cl^−^ current, which was accompanied by upregulation of the osteoclast marker genes nuclear factor of activated T cell 1 (*NFATc1*), acid phosphatase 5 (*Acp5*), cathepsin K (*Ctsk*) and matrix metalloprotein 9 (*Mmp9*) (Fig. 1a and Supplementary Fig. 1a). The Cl^−^ current at +160 mV increased from 717.7 ± 38.11 to 1712 ± 98.38 pA after induction for 5 days, and the characteristic of the outward-rectifying Cl^−^ current did not change (Fig. 1a). Cl^−^ homeostasis is mediated by a variety of ion channels, to identify the channel responsible for osteoclast differentiation, we measured the expression levels of Cl^−^ channels, including chloride voltage-gated channel 1–7 (*Clcn1*, *Clcn2*, *Clcn3*, *Clcn4*, *Clcn5*, *Clcn6*, *Clcn7*), *Ano1*, *Ano2*, and cystic fibrosis transmembrane conductance regulator (*Cftr)*, in osteoclasts. The results showed that the expression of *Clcn7*, *Ano1*, *Cftr*, and *Clcn4* was much higher than that of other channels in osteoclasts (Fig. 1b). Interestingly, only the expression levels of *Ano1* and *Clcn7* were increased during osteoclast differentiation (Fig. 1c, d and Supplementary Fig. 1b). To further test the effects of *Ano1*, *Clcn7*, *Cftr*, and *Clcn4* on osteoclast function, siRNA-mediated knockdown was used to decrease the levels of these genes. We found that *Ano1* siRNA significantly decreased the number of TRAP^+^ cells and that *Clcn7* siRNA had a slightly weaker inhibitory effect than *Ano1* siRNA. However, *Cftr* and *Clcn4* siRNA had no effect on osteoclast activity (Supplementary Fig. 1c, d). To determine the effect of Ano1 on Cl^−^ current in osteoclasts, we treated osteoclasts with *Clcn7* siRNA or CaCC blocker CaCC_inh_-A01 (A01, 20 μM). A01 blocked the pore of Ano1 and further caused the collapse of the pore. The collapse of the pore is associated with α4 main chain motion, binding of A01 leads to α4 moving to the direction of pore, which is an allosteric mode that is not conducive to pore opening^16^. Whole-cell patch clamp recording showed that the Cl^−^ current could not be completely suppressed in osteoclasts after treatment with *Clcn7* siRNA (Supplementary Fig. 1e). However, the Cl^−^ current almost disappeared when osteoclasts were cotreated with *Clcn7* siRNA and A01 (Supplementary Fig. 1e). These data suggested that Ano1 is an important contributor to the Cl^−^ current in osteoclasts. To investigate the role of Ano1 in regulating osteoclast activity and function, we treated osteoclasts with A01 or benzbromarone (10 μM). Benzbromarone not only blocks Ano1 channels, but also works in part through membrane hyperpolarization and attenuation of calcium flux at the plasma membrane^17,18^. The results showed that these agents substantially reduced the number of TRAP^+^ cells (from 99.00 ± 2 to 59 ± 3 for A01 treatment and from 118 ± 4 to 117 ± 4 for benzbromarone treatment) (Fig. 1e, f) and significantly downregulated the expression of the osteoclast marker genes *NFATc1*, *Acp5*, *Ctsk*, and *Mmp9* (Supplementary Fig. 1f, g). These data suggested that Ano1 mediates the differentiation and function of osteoclasts. It is known that the conserved amino acid residues E702 and E705 are two key sites for Ca^2+^-dependent channel activation. We found that the regulation of Ano1 on osteoclast activity was also dependent on its binding with Ca^2+^. It is wild-type Ano1 that can rescue reduced osteoclast activities caused by Ano1 deficiency, but not Ano1 with Ca^2+^-binding sites mutants (Fig. 1g-i).

### Ano1 deficiency in osteoclasts increases bone mass

**Fig. 2:**
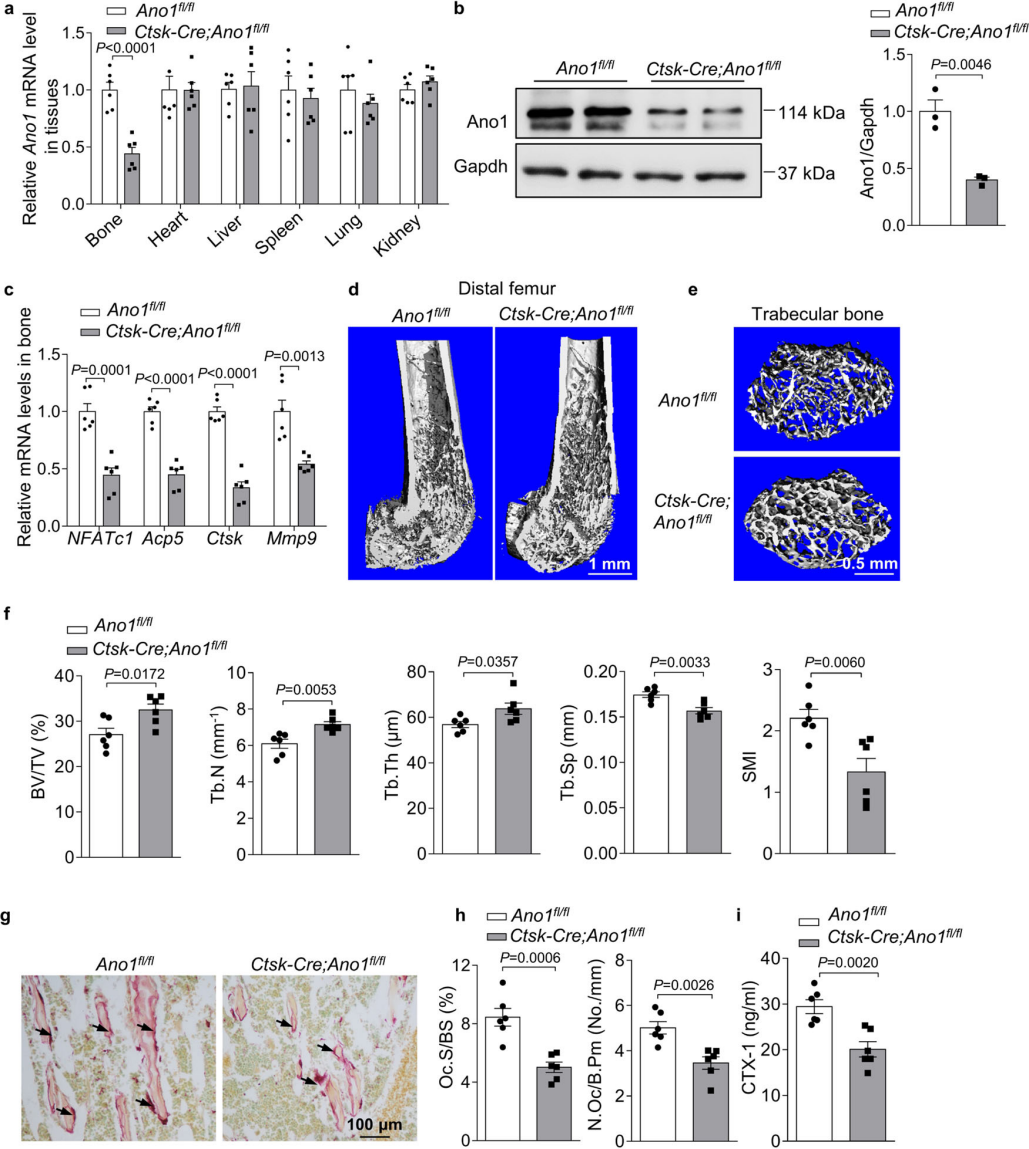
Osteoclast-specific *Ano1* knockout increases bone mass. **a** QRT-PCR analysis of *Ano1* mRNA level in bone and other tissues from 2 month-old *Ano1*^*fl/fl*^ and osteoclast-specific *Ano1* knockout (*Ctsk-Cre;Ano1*^*fl/fl*^*)* mice (*n* = 6). *Ano1* mRNA level in all tissues was normalized to *Ano1*^*fl/fl*^ mice. **b** Western blot analysis of Ano1 protein level in bone tissues from *Ano1*^*fl/fl*^ and *Ctsk-Cre;Ano1*^*fl/fl*^ mice (left). The quantification of Ano1 protein level in bone from two groups (right) (*n* = 3). **c** QRT-PCR analysis of *NFATc1, Acp5, Ctsk and Mmp9* mRNA levels in bone tissues from *Ano1*^*fl/fl*^ and *Ctsk-Cre;Ano1*^*fl/fl*^ mice (*n* = 6). **d** Representative images showing three-dimensional distal femurs trabecular architecture by micro-CT reconstruction from *Ano1*^*fl/fl*^ and *Ctsk-Cre;Ano1*^*fl/fl*^ mice at 8 weeks old (*n* = 6). Scale bar, 1 mm. **e** Representative images showing three-dimensional trabecular architecture by micro-CT reconstruction at the distal femurs from *Ano1*^*fl/fl*^ and *Ctsk-Cre;Ano1*^*fl/fl*^ male mice at 2 months old. Representative images of six independent tissue in each group. Scale bar, 0.5 mm. **f** Micro-CT measurements for BV/TV, Tb.N, Tb.Th, Tb.Sp, and SMI at the distal femurs of mice (*n* = 6). BV/TV, ratio of bone volume to tissue volume; Tb.N, trabecular number; Tb.Th, trabecular thickness; Tb.Sp, trabecular separation; SMI, structure model index. **g** Representative images of TRAP staining of the proximal tibia of mice (*n* = 6 tissues). Scale bar, 100 μm. **h** Histomorphometric analysis of the images for number of osteoclasts per bone perimeter (N.Oc/B.Pm) and osteoclast surface per bone surface (Oc.S/BS) (*n* = 6). **i** ELISA analysis of CTX-1 protein level in the serum from *Ano1*^*fl/fl*^ and *Ctsk-Cre;Ano1*^*fl/fl*^ mice (*n* = 6). All data are the mean ± s.e.m. Statistical analysis for comparison of two groups was performed using two-tailed unpaired Student’s *t*-test. Source data are provided as a Source Data file.

To investigate the function of Ano1 in osteoclasts in vivo, we generated osteoclast-specific conditional Ano1 knockout (*Ctsk-Cre;Ano1*^*fl/fl*^) mice by crossing *Ctsk-Cre* mice with *Ano1*^fl/fl^ mice^19^. We detected the mRNA and protein levels of Ano1 in bone tissue derived from 2-month-old *Ano1*^*fl/fl*^ and *Ctsk-Cre;Ano1*^*fl/fl*^ mice. *Ctsk-Cre;Ano1*^*fl/fl*^ mice showed significantly lower expression of Ano1 than *Ano1*^fl/fl^ mice in bone, but not in other tissues (Fig. 2a, b). We analyzed the expression of osteoclast marker genes, and the results showed that *NFATc1*, *Acp5*, *Ctsk* and *Mmp9* mRNA levels were significantly lower in the bone of the *Ctsk-Cre;Ano1*^*fl/fl*^ mice than in those of *Ano1*^*fl/fl*^ mice (Fig. 2c). Micro-CT analysis showed that *Ctsk-Cre;Ano1*^*fl/fl*^ mice exhibited higher bone mass than *Ano1*^*fl/fl*^ mice in femur and vertebra (Fig. 2d, e and Supplementary Fig. 2a). In the *Ctsk-Cre;Ano1*^*fl/fl*^ mice femur, the ratio of bone volume to tissue volume (BV/TV), trabecular number (Tb.N), and trabecular thickness (Tb.Th) were all significantly increased, whereas the trabecular spacing (Tb.Sp) and structure model index (SMI) were accordingly decreased (Fig. 2f). Similar results were obtained in the vertebrae, BV/TV, Tb.N, Tb.Th, were all significantly increased, SMI were accordingly decreased in the *Ctsk-Cre;Ano1*^*fl/fl*^ mice (Supplementary Fig. 2b). TRAP staining confirmed that the activity of osteoclasts along the surface of trabecular bone was lower in *Ctsk-Cre;Ano1*^*fl/fl*^ mice than in *Ano1*^*fl/fl*^ mice (Fig. 2g). Osteoclast number per bone perimeter (N.Oc/B.pm) and osteoclast surface per bone surface (Oc.S/BS) were both lower in *Ctsk-Cre;Ano1*^*fl/fl*^ mice than in *Ano1*^*fl/fl*^ mice (Fig. 2h). Accordingly, the levels of c-telopeptide of type I collagen (CTX-1) were significantly downregulated in serum derived from *Ctsk-Cre;Ano1*^*fl/fl*^ mice compared to that derived from *Ano1*^*fl/fl*^ (Fig. 2i). These results suggested that osteoclast-specific knockout of Ano1 inhibits bone resorption in the *Ctsk-Cre;Ano1*^*fl/fl*^ mice and increases bone mass.

### Osteoclast-specific *Ano1* overexpression reduces bone density

**Fig. 3:**
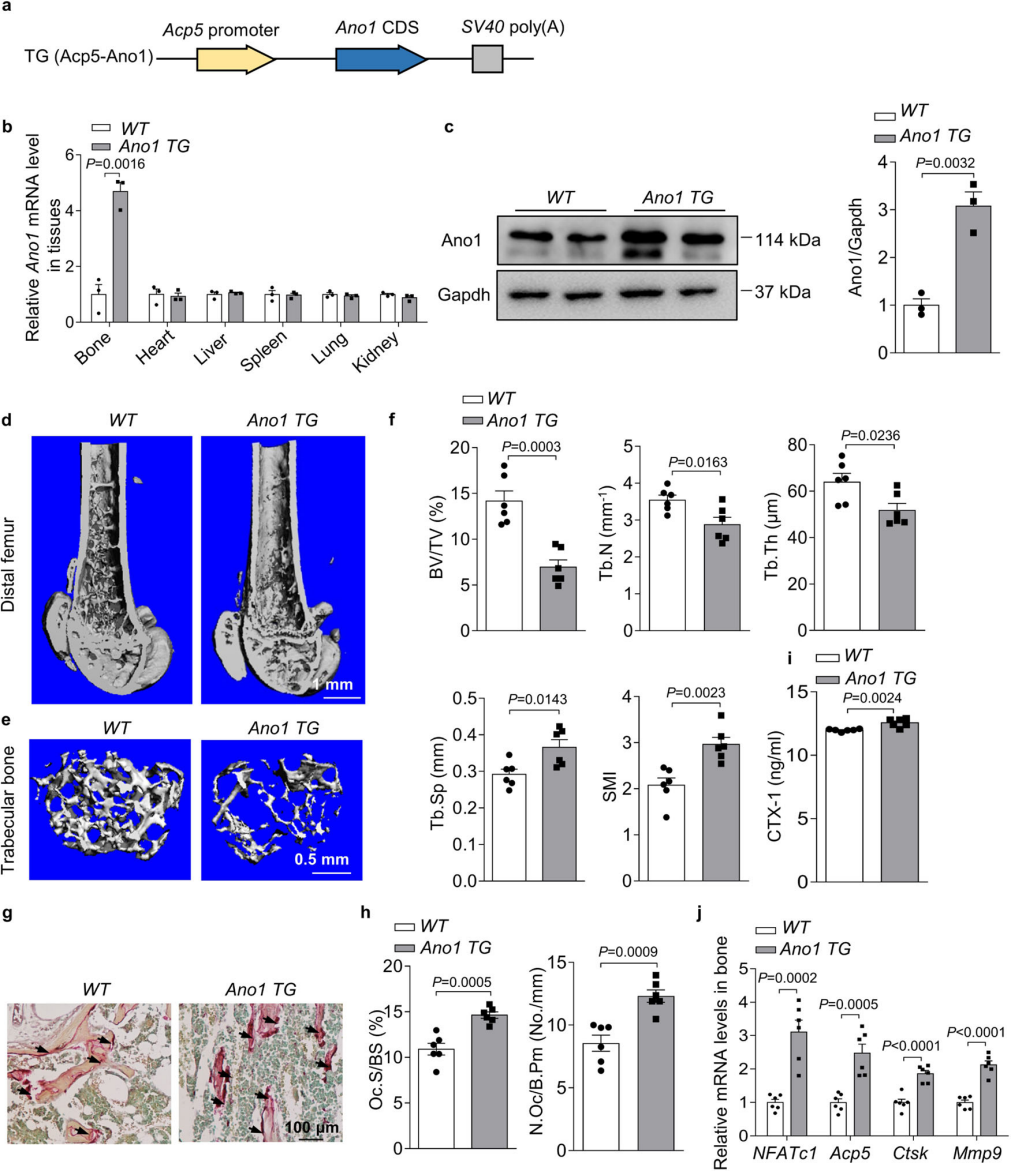
*Ano1* overexpression decreases bone mass. **a** Schematic representation of the transgenic construct used to generate Ano1 transgenic mouse lines. **b** QRT-PCR analysis of *Ano1* mRNA level in bone and other tissues from 6-month-old WT and osteoclast-specific *Ano1* over expression (*Ano1 TG*) mice (*n* = 3). *Ano1* mRNA level in all tissues was normalized to *WT* mice. **c** Western blot analysis of Ano1 protein level in bone tissues from *WT* and *Ano1 TG* mice mice (left). The quantification of Ano1 protein level in bone from two groups (right) (*n* = 3). **d** Representative images showing three-dimensional distal femurs by micro-CT reconstruction from *WT* and *Ano1 TG* female mice at 6 months old. Scale bar, 1 mm. **e** Representative images showing three-dimensional trabecular architecture by micro-CT reconstruction at the distal femurs from *WT* and *Ano1 TG* mice at 6 months old. Representative images of six independent tissue in each group. Scale bar, 0.5 mm. **f** Micro-CT measurements for BV/TV, Tb.N, Tb.Th, Tb.Sp, and SMI at the distal femurs from *WT* and *Ano1 TG* mice (*n* = 6). **g** Representative images of TRAP staining of the proximal tibia from *WT* and *Ano1 TG* mice. Scale bar, 100 μm. **h** Histomorphometric analysis of the images for number of osteoclasts per bone perimeter (N.Oc/B.Pm) and osteoclast surface per bone surface (Oc.S/BS) (*n* = 6). **i** ELISA analysis of CTX-1 protein levels in the serum from *WT* and *Ano1 TG* mice (*n* = 6). **j** QRT-PCR analysis of *NFATc1, Acp5, Ctsk*, and *Mmp9* mRNA levels in bone tissues from *WT* and *Ano1 TG* mice (*n* = 6). All data are the mean ± s.e.m. Statistical analysis for comparison of two groups was performed using two-tailed unpaired Student’s *t*-test. Source data are provided as a Source Data file.

To further investigate the role of *Ano1* overexpression in osteoclasts in vivo, we developed a construct that expresses *Ano1* under the control of the *Acp5* promoter (Fig. 3a), which has been shown to be specifically activated in osteoclasts. Subsequently, we established *Ano1 TG* mice. *Ano1 TG* mice showed significantly higher expression of *Ano1* than WT mice in the bone, but not in other tissues (Fig. 3b, c). We next carried out micro-CT analysis. In the *Ano1 TG* mice, trabecular bone mass, bone parameters including BV/TV, Tb.N, Tb.Th,Tb.Sp and SMI were not different from WT mice at 2-month-old (Supplementary Fig. 3a, b). At 4-month-old, we can see the decrease trend in the bone mass and bone parameters in *Ano1 TG* mice, although there were no significant differences (Supplementary Fig. 3c, d). In 6-month-old mice, trabecular bone mass was significantly lower than that in WT mice (Fig. 3d, e), as confirmed by the decrease in BV/TV, Tb.N, Tb.Th, and the increase in Tb.Sp and SMI (Fig. 3f). TRAP staining data showed that osteoclast activity, N.Oc/B.pm and Oc.S/BS were higher in the proximal tibiae of *Ano1 TG* mice than in those of WT mice (Fig. 3g, h). Accordingly, the level of CTX-1 was significantly higher in serum derived from *Ano1 TG* mice than in that derived from WT mice (Fig. 3i). To test the promoting effects of Ano1 on osteoclasts differentiation in vivo, we examined the expression of osteoclast marker genes in bone tissue and osteoclasts. QRT-PCR analysis showed that *NFATc1*, *Acp5*, *Ctsk*, and *Mmp9* mRNA levels were significantly higher in *Ano1 TG* mice than that in WT mice (Fig. 3j). These results suggested that Ano1 overexpression promotes osteoclast function, increases bone resorption and decreases bone mass.

### Promotional effect of Ano1 channel activity on osteoclast differentiation and function

**Fig. 4:**
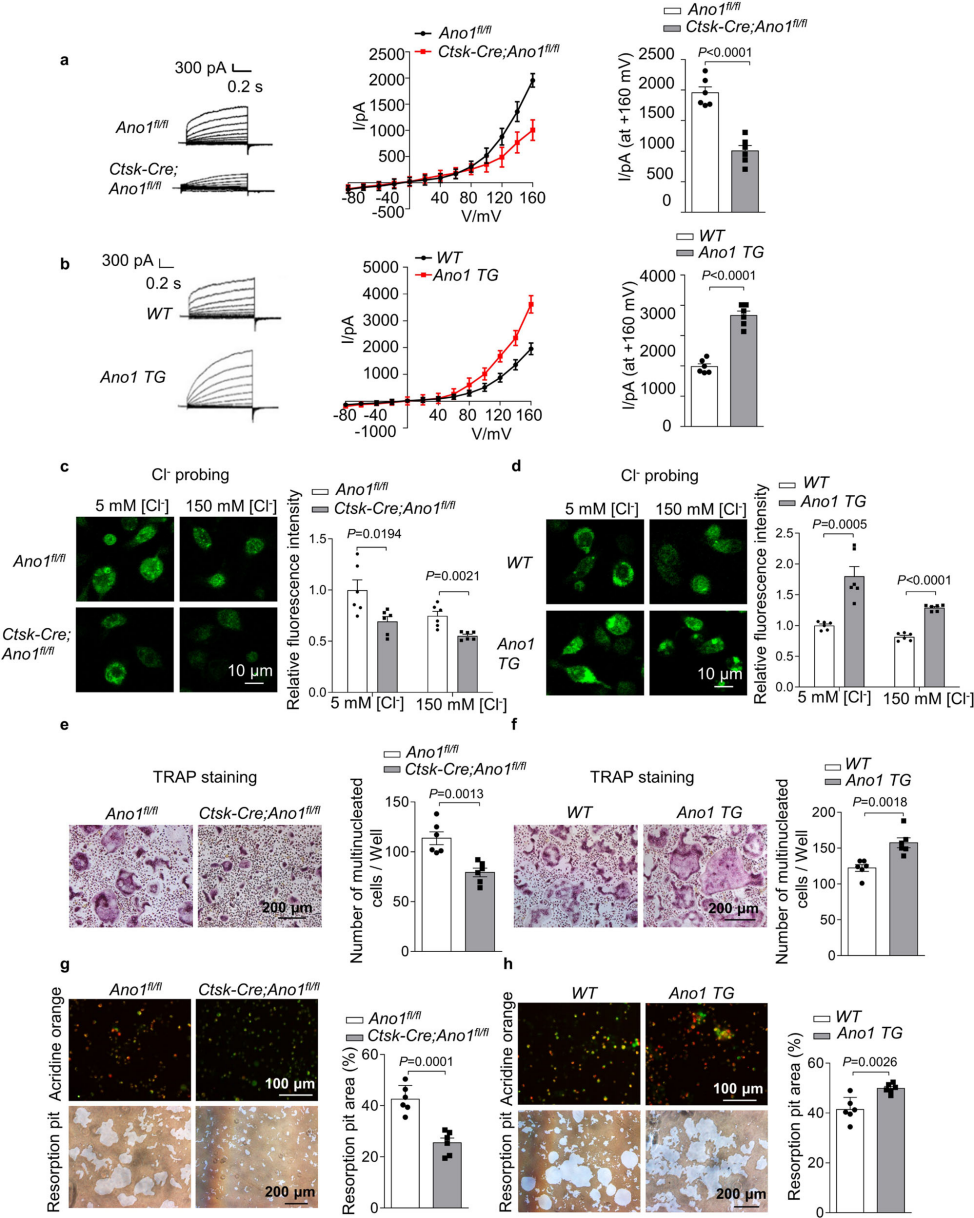
Ano1 regulates osteoclast differentiation and function via modulating intracellular chloride concentration. **a** Representative chloride currents recorded from voltage ramps from −80 to +160 mV in whole-cell patch-clamp of RANKL-induced osteoclasts from *Ano1*^*fl/fl*^ and *Ctsk-Cre;Ano1*^*fl/fl*^ mice for 5 days. **b** Representative chloride currents recorded from voltage ramps from −80 to +160 mV in whole-cell patch-clamp of RANKL-induced osteoclasts from *WT* and *Ano1 TG* mice for 5 days. **c**, **d** Measurement of intracellular chloride concentration in RANKL-inducted osteoclasts. **c** Representative fluorescent images of RANKL-induced osteoclasts from *Ano1*^*fl/fl*^ and *Ctsk-Cre;Ano1*^*fl/fl*^ mice for 5 days (left). The relative fluorescence intensity of RANKL-induced osteoclasts (right) (*n* = 6 well). All the cells were stained with 5 mM MQAE. Scale bar, 10 μm. **d** Representative fluorescent images of RANKL-induced osteoclasts from *WT* and *Ano1 TG* mice for 5 days (left). The relative fluorescence intensity of RANKL-induced BMMs cells (right) (*n* = 6 well). All the cells were stained with 5 mM MQAE. Scale bar, 10 μm. **e** Representative images of TRAP staining in RANKL-induced osteoclasts from *Ano1*^*fl/fl*^ and *Ctsk-Cre;Ano1*^*fl/fl*^ mice for 5 days (left). Quantification of the number of multinucleated cells per well (right). **f** Representative images of TRAP staining in RANKL-induced osteoclasts from *WT* and *Ano1 TG* mice for 5 days (left). Quantification of the number of multinucleated cells per well (right). **g** Representative images of acridine orange staining and resorption pits in RANKL-induced osteoclasts from *Ano1*^*fl/fl*^ and *Ctsk-Cre;Ano1*^*fl/fl*^ mice for 5 days (left). Quantification of the resorption pit area (right). **h** Representative images of acridine orange staining and resorption pits in RANKL-induced osteoclasts from *WT* and *Ano1 TG* mice for 5 days (left). Quantification of the resorption pit area (right). All data are the mean ± s.e.m. from three independent experiments. Two-tailed unpaired Student’s *t*-test was used for statistical evaluations of two group comparisons. Source data are provided as a Source Data file.

To investigate the role of Ano1 channel activity in osteoclast function, we measured the changes of Cl^−^ current in osteoclast. First, we detected the mRNA and protein levels of Ano1 in osteoclasts isolated from *Ano1*^*fl/fl*^ and *Ctsk-Cre;Ano1*^*fl/fl*^ mice. *Ctsk-Cre;Ano1*^*fl/fl*^ osteoclasts showed significantly lower expression of Ano1 than *Ano1*^fl/fl^ osteoclasts (Supplementary Fig. 4a, b). Accordingly, the expression of functional markers of osteoclast was significantly inhibited (Supplementary Fig. 4c). Then, we found that *Ano1* knockout obviously attenuated the gradually increase in the Cl^−^ currents. The Cl^−^ current in osteoclasts from *Ano1*^*fl/fl*^ mice at +160 mV was 1956 ± 95.56 pA, whereas that in osteoclasts from *Ctsk-Cre;Ano1*^*fl/fl*^ mice was reduced to 1005 ± 87.53 pA (Fig. 4a). In contrast, *Ano1* overexpression increased the Cl^−^ current and upregulated the expression of functional markers of osteoclast (Fig. 4b, Supplementary Fig. 4d–f). The Cl^−^ current in *Ano1 TG* osteoclasts was 3665 ± 144.2 pA, which was two times that in WT osteoclasts (Fig. 4b). The regulation of Ano1 on Cl^−^ currents prompted us to test the changes of the cytoplasmic Cl^−^ level in osteoclasts. Here, we use a Cl^−^ sensor (the fluorescent probe, MQAE), the fluorescence intensity of this sensor is inversely related to the intracellular Cl^−^ concentration, and a decrease in the fluorescence intensity correlates with an increase in the cytoplasmic Cl^−^ level^15^. We observed that *Ctsk-Cre;Ano1*^*fl/fl*^ osteoclast showed lower fluorescence intensity of Cl^−^ sensor than that in *Ano1*^*fl/fl*^ osteoclast (Fig. 4c). Bathing in low Cl^−^ solution (5 mM Cl^−^ and 145 mM gluconate), fluorescence intensity in *Ctsk-Cre;Ano1*^*fl/fl*^ osteoclast was 69.3% that of *Ano1*^*fl/fl*^ osteoclast. Bathing in high Cl^−^ solution (150 mM Cl^−^), fluorescence intensity in *Ctsk-Cre;Ano1*^*fl/fl*^ osteoclast was 74% that of *Ano1*^*fl/fl*^ osteoclast. However, the fluorescence intensity of the Cl^−^ sensor in *Ano1 TG* osteoclasts was higher than that in WT osteoclasts (Fig. 4d). Bathing in low Cl^−^ solution, fluorescence intensity in *Ano1 TG* osteoclast was 1.8 fold that of WT osteoclast. Bathing in high Cl^−^ solution, fluorescence intensity in *Ano1 TG* osteoclast was 1.3 fold that of WT osteoclast. These results indicated the important role of Ano1 in controlling intracellular Cl^−^ homeostasis.

Adequate intracellular Cl^−^ levels are required for the maintenance of membrane phosphoinositide homeostasis and remodeling, which are involved in the regulation of key signaling molecules for osteoclast morphology and function. Ex vivo TRAP staining experiments revealed that there were markedly fewer TRAP^+^ multinucleated osteoclasts derived from *Ctsk-Cre;Ano1*^*fl/fl*^ osteoclasts and substantially more TRAP^+^ multinucleated osteoclasts derived from *Ano1 TG* mice than that from the control mice (Fig. 4e, f). Acidification is fully dependent on and proportional to the Cl^−^ concentration. Secretion of protons into the isolated extracellular compartment is essential for osteoclast bone resorption. We found that osteoclasts from *Ctsk-Cre;Ano1*^*fl/fl*^ mice showed lower extracellular acidification than osteoclasts from *Ano1*^*fl/fl*^ mice and that osteoclasts from *Ano1 TG* mice exhibited the opposite change (Fig. 4g, h). Accordingly, the area of resorption pit of *Ctsk-Cre;Ano1*^*fl/fl*^ osteoclasts were 59.8% that of *Ano1*^*fl/fl*^ osteoclasts and *Ano1 TG* osteoclasts were increased by more than 1.2 fold compared with the WT osteoclasts (Fig. 4g, h).

### The crosstalk between Ano1 and RANKL signaling in osteoclasts

**Fig. 5:**
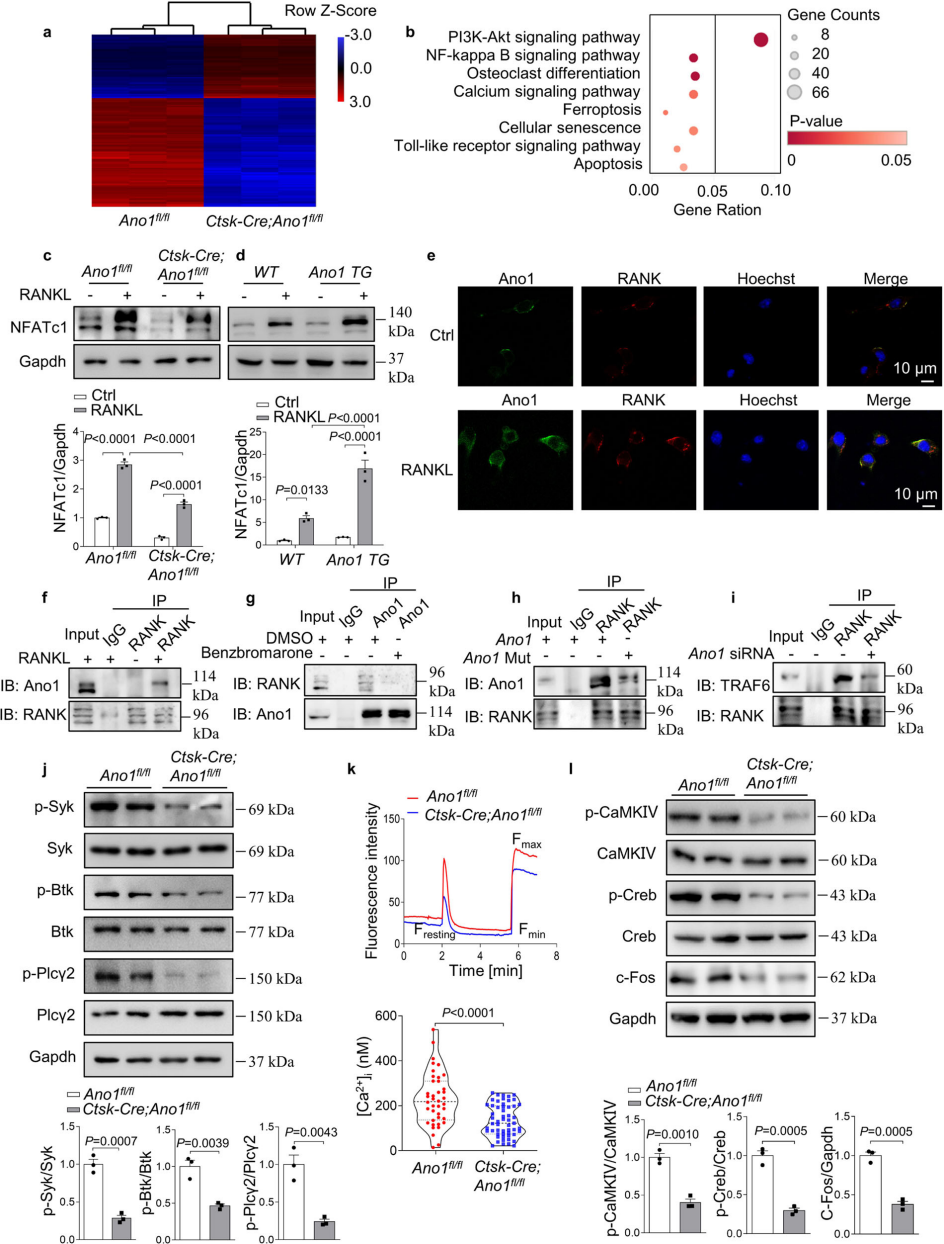
Calcium-activated chloride channel Ano1 promotes RANKL signaling via its activity-dependent interaction with RANK. **a** Microarray assays were performed in osteoclasts originated from *Ano1*^*fl/fl*^ (*n* = 3) and *Ctsk-Cre;Ano1*^*fl/fl*^ (*n* = 3) mice. The relative mRNA expression is depicted according to the color, red indicates upregulation and blue indicates downregulation. Shown are the mRNAs that changed more than 2 folds. **b** Pathway enrichment analysis (Gene Analytics) for genes expressed differentially between *Ano1*^*fl/fl*^ and *Ctsk-Cre;Ano1*^*fl/fl*^ osteoclasts. **c** Western blot analysis of NFATc1 protein level in *Ano1*^*fl/fl*^ and *Ctsk-Cre;Ano1*^*fl/fl*^ osteoclasts after treatment with or without RANKL mice (top). The quantification of NFATc1 protein level in osteoclasts (below). **d** Western blot analysis of NFATc1 protein level in *WT* and *Ano1 TG* osteoclasts after treatment with or without RANKL (top). The quantification of NFATc1 protein level in osteoclasts (below). **e** Immunofluorescence of Ano1 (green) and RANK (red) in osteoclasts treated with or without RANKL by confocal microscopy. Representative images are shown. Scale bar, 10 μm. **f** Coimmunoprecipitation of Ano1 and RANK in osteoclasts after treatment with or without RANKL. **g** Coimmunoprecipitation of Ano1 and RANK in RANKL-induced osteoclasts after treatment with Ano1 inhibitor Benzbromarone (10 μM). **h** Coimmunoprecipitation of Ano1 and RANK in RANKL-induced osteoclasts after overexpression of *Ano1* or *Ano1* with E702Q and E705Q mutants. **i** Coimmunoprecipitation of RANK and TRAF6 in RANKL-induced osteoclasts transfected with *Ano1* siRNA or its negative control. **j** Western blot analysis of the phosphorylation levels of Syk, Btk, and Plcγ2 in osteoclasts (top). The quantification of the phosphorylation level of Syk, Btk, and Plcγ2 in osteoclasts (below). **k** Resting [Ca2+]_i_ in osteoclasts from *Ano1*^*fl/fl*^ mice and *Ctsk-Cre;Ano1*^*fl/fl*^ mice, *n* = 40 (*Ano1*^*fl/fl*^) and *n* = 55 (*Ctsk-Cre;Ano1*^*fl/fl*^) cells pooled from three independent experiments. **l** Western blot analysis of p-CaMKIV, p-Creb, and c-Fos protein levels in osteoclasts (top). The quantification of p-CaMKIV, p-Creb, and c-Fos protein levels in osteoclasts (below). All data are the mean ± s.e.m. from three independent experiments. Two-tailed unpaired Student’s *t*-test was used for statistical evaluations of two group comparisons. Statistical analysis with more than two groups was performed with two-way analysis of variance (ANOVA) with Šídák post-hoc test to determine group differences. Source data are provided as a Source Data file.

To identify pathways and mechanisms regulated by Ano1 during osteoclastogenesis, we performed unbiased transcriptomic analysis using microarray of osteoclasts derived from *Ano1*^*fl/fl*^ mice and *Ctsk-Cre;Ano1*^*fl/fl*^ mice. A total of 2513 genes were upregulated (>2-fold) and 2814 genes were downregulated (>2-fold) in osteoclasts from *Ctsk-Cre;Ano1*^*fl/fl*^ mice compared to those from *Ano1*^*fl/fl*^ mice (Fig. 5a). Notably, pathway enrichment analysis of these genes revealed significant enrichment for the PI3K-Akt, NFκB, and calcium signaling pathways, which are essential for osteoclast differentiation (Fig. 5b). All of these pathways are key downstream regulators of the RANKL–RANK signaling pathway, which is essential for osteoclastogenesis. The expression of NFATc1, the master regulator of osteoclast differentiation, was markedly affected by Ano1, as demonstrated by the significant reduction in the NFATc1 level in osteoclasts from *Ctsk-Cre;Ano1*^*fl/fl*^ mice and the substantial increased in the NFATc1 level in osteoclasts from *Ano1 TG* mice compared to those from control mice under RANKL stimulation (Fig. 5c, d).

The above results suggested that Ano1, as a transmembrane protein, functions upstream of RANKL–RANK pathway. This prompted us to test whether there existed interaction between Ano1 and RANK. Immunofluorescence results show that Ano1 is co-localized with RANK at the plasma membrane of osteoclasts induced by RANKL (Fig. 5e). The coimmunoprecipitation demonstrated that RANK and Ano1 interact strongly in RANKL-treated osteoclasts but do not interact in the absence of RANKL stimulation (Fig. 5f). Interestingly, benzbromarone, an Ano1 inhibitor, obviously attenuated their interaction (Fig. 5g). We also found that the interaction between Ano1 and RANK was attenuated when E702Q and E705Q mutations were introduced into Ano1 (Fig. 5h). This suggested that the interaction between Ano1 and RANK is also Ca^2+^ dependent.

Next, we tested whether the interaction between Ano1 and RANK can regulate its binding to tumor necrosis factor (TNF) receptor-associated factor 6 (TRAF6), which is the most important adaptor for RANKL–RANK-induced osteoclastogenesis. The coimmunoprecipitation assay demonstrated that *Ano1* siRNA severely impaired the association between RANK and TRAF6 (Fig. 5i). RANKL–RANK signaling activates spleen tyrosine kinase (Syk), recruits both Bruton’s tyrosine kinase (Btk) kinases and phospholipase C γ2 (PLCγ2) to form the osteoclastogenic signaling complex and leads to an increase in [Ca^2+^]_i_^20,21^. Thus, we examined the changes in downstream signaling following *Ano1* knockout or overexpression in osteoclast. The results demonstrated that the phosphorylation of Syk, Btk and PLCγ2 was significantly suppressed in osteoclasts from *Ctsk-Cre;Ano1*^*fl/fl*^ mice than those from *Ano1*^*fl/fl*^ mice (Fig. 5j). Phosphorylated PLCγ2 catalyzes the hydrolysis of PtdIns(4,5)*P*_2_ to produce inositol (1, 4, 5)-trisphosphate (Ins(1, 4, 5)*P*_3;_ IP3) and diacylglycerol, which stimulates the release of Ca^2+^ from the endoplasmic reticulum. Here, we observed that the [Ca^2+^]_i_ was significantly lower in osteoclasts from *Ctsk-Cre;Ano1*^*fl/fl*^ mice (Fig. 5k) and that decrease in the [Ca^2+^]_i_ reduced CaMKs. CaMKIV-Creb is crucial for osteoclast differentiation and function^22^. We found that the phosphorylation of CaMKIV and Creb was significantly reduced in osteoclasts from *Ctsk-Cre;Ano1*^*fl/fl*^ mice than in those from *Ano1*^*fl/fl*^ mice. Accordingly, the expression of c-Fos, the key transcription factor for the induction of NFATc1, was substantially inhibited in osteoclasts from *Ctsk-Cre;Ano1*^*fl/fl*^ osteoclasts compared to those from *Ano1*^*fl/fl*^ mice (Fig. 5l). To further confirm the role of Ano1 in this process, we detected changes in the above parameters in osteoclasts from *Ano1 TG* mice. Our analysis revealed that under RANKL stimulation, Ano1 overexpression promoted the phosphorylation of Syk, Btk, and PLCγ2 (Supplementary Fig. 5a), increased the [Ca^2+^]_i_ (Supplementary Fig. 5b), activated CaMKIV and Creb activity and induced c-Fos expression (Supplementary Fig. 5c). These results indicated that Ano1 is required for RANKL-mediated CaMK-Creb activation during osteoclastogenesis.

In addition, we detected the effect of Ano1 on other pathways downstream of the RANKL–RANK–TRAF6-dependent axis, including the mitogen-activated protein kinase (MAPK), nuclear factor kappa B (NFκB), and serine/threonine kinase (AKT) pathways^23^. The results showed that p-AKT, p-JNK, p-p38 and p-ERK levels were all lower in osteoclasts from *Ctsk-Cre;Ano1*^*fl/fl*^ mice than in those from *Ano1*^*fl/fl*^ mice and higher in osteoclasts from *Ano1 TG* mice than in those from WT mice (Supplementary Fig. 5d, e). These results indicated the critical role of Ano1 in the activation of RANKL-RANK-TRAF6 signaling during osteoclast differentiation.

### The role of Ano1 in OVX-induced bone loss

**Fig. 6:**
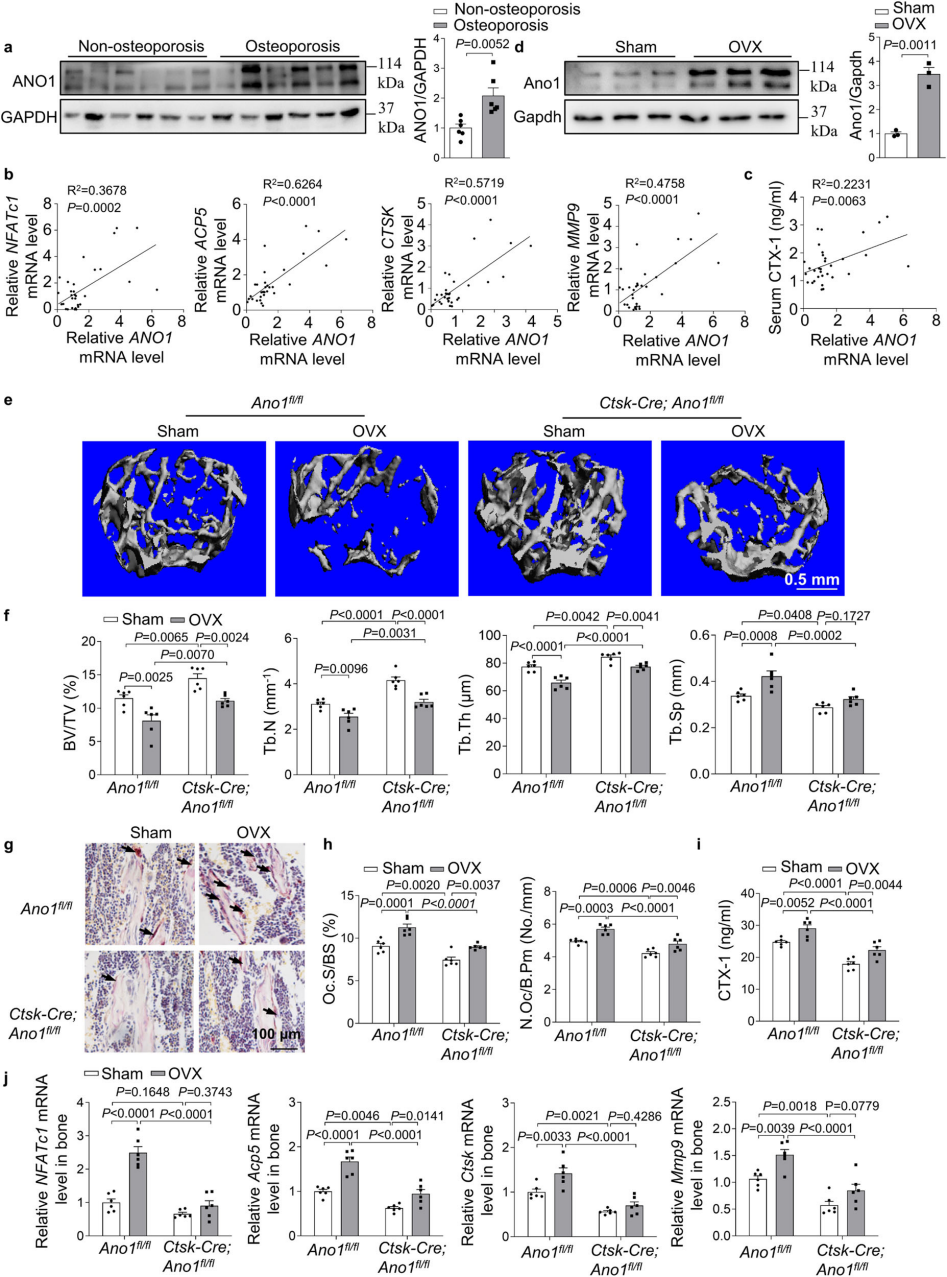
*Ano1* knockout in osteoclast inhibits OVX-induced bone loss. **a** Western blot analysis of ANO1 protein level in human bone specimens from two groups (left). The quantification of ANO1 protein level in human bone specimens from two groups (right). Non-osteoporosis group (*T*-score > −2.5), *n* = 6, and Osteoporosis group (*T*-score ≤ −2.5), *n* = 6. **b** Correlation analysis between *ANO1* mRNA level and *NFATc1*, *ACP5*, *CTSK* and MMP9 mRNA levels in human bone specimens (*n* = 32). **c** Correlation analysis between ANO1 mRNA level in bone tissues and β-CTX level in human serum (*n* = 32). **d** Western blot analysis of Ano1 protein level in bone tissues with Sham or ovariectomized (OVX) treatment (left). The quantification of Ano1 protein level in bone tissues with Sham or OVX treatment (right) (*n* = 3). **e** Representative images showing three-dimensional trabecular architecture as determined by micro-CT reconstruction of the distal femurs from the groups of mice indicated. Representative images of six independent tissue in each group. Scale bar, 0.5 mm. **f** Micro-CT measurements for BV/TV, Tb.N, Tb.Th and Tb.Sp in the distal femurs. *n* = 6 for each group. **g** Representative images of TRAP staining of the proximal tibia. Scale bar, 100 μm. *n* = 6 for each group. **h** Histomorphometry analysis of the images for number of osteoclasts per bone perimeter (N.Oc/B.Pm) and osteoclast surface per bone surface (Oc.S/BS). *n* = 6 for each group. **i** ELISA analysis of CTX-1 protein level in serum. *n* = 6 for each group. **j** QRT-PCR analysis of *NFATc1*, *Acp5*, *Ctsk*, and *Mmp9* mRNA levels in bone tissues. *n* = 6 for each group. All data are the mean ± s.e.m. Two-tailed unpaired Student’s *t*-test was used for statistical evaluations of two group comparisons. Statistical analysis with more than two groups was performed with two-way analysis of variance (ANOVA) with Šídák post-hoc test to determine group differences. Source data are provided as a Source Data file.

The close relationship between ANO1 expression and function in osteoclasts prompted us to explore the pathological role of ANO1 in human osteoporosis. We collected bone specimens from 32 patients with fractures (Supplementary Table 1). The protein level of ANO1 in osteoporosis patients (*T* ≤ −2.5) was significantly higher than that in non-osteoporosis patients (*T* > −2.5) (Fig. 6a). Furthermore, the expression of *ANO1* was positively correlated with the expression of osteoclast marker genes, including *NFATc1*, *ACP5*, *CTSK*, and *MMP9*, in these human samples (Fig. 6b). We also analyzed the correlation between ANO1 mRNA level and serum β-CTX protein level. The results showed that the expression of ANO1 was positively correlated with serum β-CTX protein level (Fig. 6c). Thus, we further explored the change of Ano1 in bone tissue of mouse models. We consistently found that high Ano1 protein level in bone tissue in OVX mice (Fig. 6d). To test whether Ano1 can be as a potential therapeutic target to prevent age-related bone loss, OVX or sham surgeries were performed in 12-week old *Ano1*^*fl/fl*^ and *Ctsk-Cre;Ano1*^*fl/fl*^ mice. As predicted, *Ano1*^*fl/fl*^ mice exhibited marked bone loss, as revealed by low bone mass, BV/TV, Tb.N and Tb.Th, increased Tb.Sp (Fig. 6e, f). OVX treated *Ctsk-Cre;Ano1*^*fl/fl*^ mice revealed increased bone mass, BV/TV, Tb.N, Tb.Th as well as decreased Tb.Sp compared with OVX treated *Ano1*^*fl/fl*^ mice (Fig. 6e, f). We observed the corresponding change in the osteoclast function. OVX-induced increase of TRAP staining, N.Oc/B.pm and Oc.S/BS in bone tissues (Fig. 6g, h), serum CTX-1 level (Fig. 6i), as well as the expression of the marker genes of osteoclasts (Fig. 6j), were specifically observed in the *Ano1*^*fl/fl*^ mice, but not in the *Ctsk-Cre;Ano1*^*fl/fl*^ mice. These results suggested that Ano1 plays a crucial role in regulating bone resorption through promoting osteoclast activity. A reduction in Ano1 levels can protect against OVX-induced bone loss. These data suggested that Ano1 is a potential therapeutic target for osteoporosis.

## Discussion

**Fig. 7:**
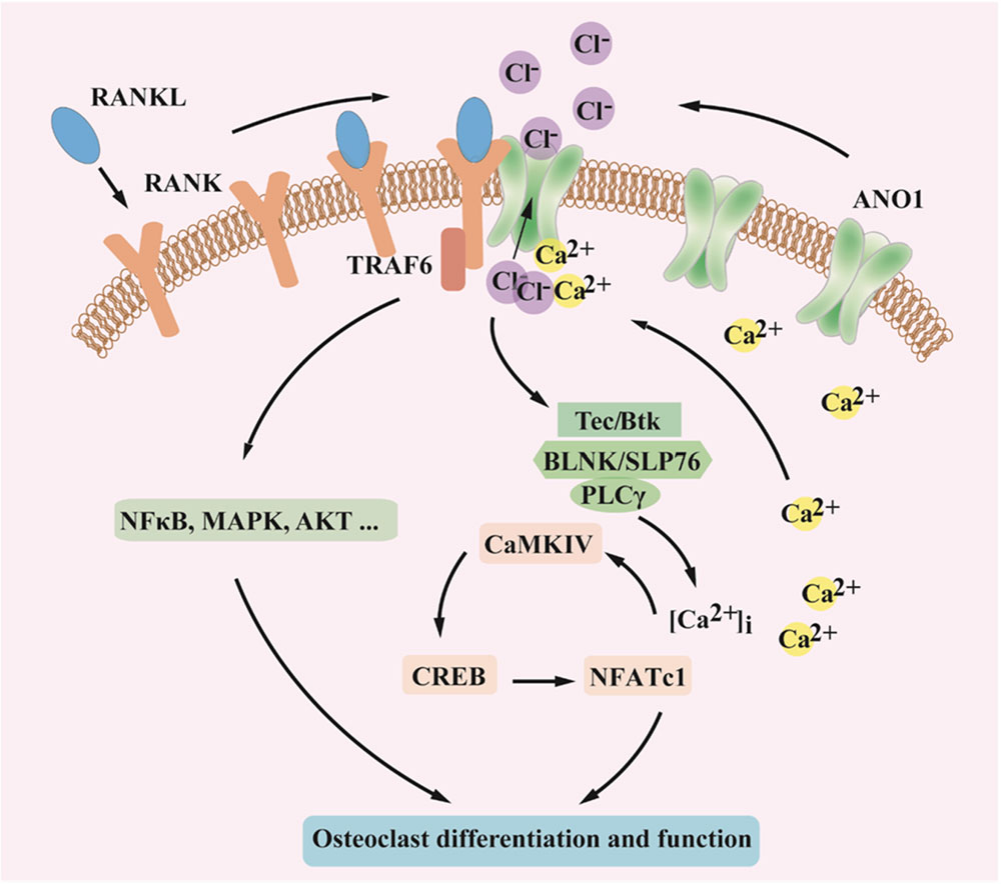
Schematic model depicting the role of ANO1-mediated osteoclast differentiation and function. Under RANKL stimulation, the interaction between Ano1 and RANK was enhanced, and RANK downstream signaling pathways was activated, which promoted the Ano1 channel activity and enhanced its interaction with RANK.

In this study, we first identified the unique role of Ano1 in osteoclastogenesis and bone resorption. We verified the importance of Ano1 for osteoclast differentiation and bone resorption with osteoclast-specific knockout and transgenic mice. Osteoclast-specific knockout of *Ano1* led to reduced osteoclast activity, increased bone density, and resistance to OVX-induced bone loss. Upregulation of Ano1, as a calcium-activated chloride channel, enhanced the Cl^−^ currents during osteoclastogenesis, promoted Cl^−^ efflux and H^+^ secretion, and increased osteoclast activity. In addition, under RANKL stimulation, the expression of Ano1 was increased, and the PLCγ-mediated calcium signal was activated, which promoted the Ano1 channel activity and enhanced its interaction with RANK. The interaction between Ano1 and RANK further promoted RANKL–RANK signaling pathway, which formed a forward feedback loop (Fig. 7). These findings indicate that Ano1 plays an essential role in osteoclast function and is a promising therapeutic target for bone loss.

Ano1 is a CaCC that is expressed on secretory epithelia, smooth muscles, sensory neurons, and hepatocytes and is found in multiple types of cancers^24–28^. It participates in various physiological processes including epithelial fluid secretion, gut motility, exocrine gland secretion, renal function, smooth muscle contraction, nociception, and metabolic regulation^6–9,29–33^. An increasing number of studies have revealed that Ano1 expression is upregulated in many diseases, including cancers, hypertension, cystic fibrosis, nonalcoholic fatty liver disease, chronic rhinosinusitis, asthma, diarrhea, gastroparesis, and polycystic kidney disease^7,27,34–40^. However, the role of Ano1 in osteoporosis and bone remodeling has been unknown. Here, we determined that Ano1 is present in osteoclasts and plays an essential role in osteoclastogenesis and bone resorption. Ano1 levels were obviously elevated during osteoclastogenesis and in the bone tissues of osteoporosis patients, and were positively correlated with osteoclast activity.

The microarray results demonstrated that Ano1 regulates all RANKL–RANK downstream pathways, including the PI3K-Akt, NF-κB, and calcium signaling pathways. RANKL–RANK causes Src-mediated Syk phosphorylation and activation, and Syk activation evokes Ca^2+^ release from the endoplasmic reticulum via activation of PLCγ, which leads to the binding of IP3 to IP3 receptors located on the endoplasmic reticulum^20,41^. Ano1 can strongly promote this process, and increases in the [Ca^2+^]_i_ and Ca^2+^-activated CaMK has dual effects. Increased [Ca^2+^]_i_ and Ca^2+^-activated CaMK promoted the expression of the osteoclast master regulator NFATc1 via phosphorylation of Creb and c-Fos. On the other hand, increased [Ca^2+^]_i_ and Ca^2+^-activated CaMK bound to Ano1 and increased its channel activity. We have identified that Ano1 was mainly distributed in the osteoclast membrane, and co-localized with RANK. Activated Ano1 exhibited a greater ability to bind to RANK and in turn promoted downstream signaling pathways. Ano1 interacted with RANK in osteoclasts, and this interaction was obviously enhanced under RANKL treatment. The Ano1/RANK interaction was found to be dependent on Ano1 ion channel activity, and an Ano1 inhibitor or mutation of Ca^2+^ binding sites significantly attenuated the interaction between Ano1 and RANK. The Ano1 has multiple open and closed states dependent on intracellular Ca^2+^. When bound with Ca^2+^, Ano1 channels adopt open state^42,43^. Given the architecture features of CACC channel, we speculate that Ano1 can interact with RANK in an open state when the channel is activated, but not in the closed state. The interaction between RANK and Ano1 potentiated the binding of RANK with TRAF6, which is an intermediate signaling protein. Downstream signaling pathways mediated by TRAF6, including the NF-κB, MAPK kinase and Akt pathways were all attenuated in *Ano1* knockout osteoclasts. RANKL signaling upregulated Ano1 protein level, and Ano1 promoted RANKL-RANK signaling. Ano1 and RANK form a functional complex, and establish a functional and regulatory link, which jointly promotes osteoclastogenesis and function.

It has been reported that Ano1 can regulate the distribution and clustering of PIP_2_ at the plasma membrane in epithelial morphogenesis^44^. PLC can cleave PIP_2_ into IP_3_, which can activate IP_3_R and lead to the release of Ca^2+^ from the endoplasmic reticulum. Here, we also observed that PLC was activated and the [Ca^2+^]_i_ was increased. Whether the lipid distribution in the osteoclast membrane is changed or Ano1 interacts with IP_3_R in osteoclasts needs to be further investigated in the future. Based on our results, we speculate that ion conduction through Ano1 and the physical interaction between Ano1 and its membrane receptor contribute to osteoclast differentiation and function.

Cl^−^ is one of the most widely distributed anions in the human body. An excessive Cl^−^ concentration in the human body leads to accelerated Ca^2+^ metabolism, osteoporosis, imbalance of cell membrane osmotic pressure, and electrolyte imbalance in the body^45,46^. Ano1 is a Cl^−^ channel, and its function in Cl^−^ transport contributes to its role in bone resorption. Here, our results demonstrated that Ano1 mediated Cl^−^ conduction is also essential for its role in osteoclastogenesis, as the Ano1 blocker benzbromarone and CaCCinh-A01 significantly inhibit osteoclast differentiation. The I/V curves of Ano1 show at membrane voltages around −40 mV. Although the inward Cl^−^ current of Ano1 is small, it does not indicate that Ano1 is unimportant. In the physiological conditions, Ano1 ion channel maintains stable cell membrane potential and osmotic pressure by mediating chloride ion flow. Only a small flow of Cl^−^ is enough to maintain cellular homeostasis of the osteoclast cell which belongs to the unexcitable cells. Cytoplasmic Cl^−^ level is dynamically regulated by Cl^−^ channels and transporters. In our study, we observed increased intracellular Cl^−^ in *Ano1* knockout osteoclasts, which was accompanied by reductions in proton secretion, fusion of osteoclast progenitor cells and bone resorption. The role of polyphosphoinositide distribution and function in this process needs to be further explored.

In this study, Ano1 was found to be a distinct Cl^−^ channel than CLC7. Ano1 functions an essential factor for osteoclast differentiation and function. CLC7 is a late endosomal/lysosomal Cl^−^ channel that is highly expressed in osteoclasts, in which it can be inserted into the ruffled border. Mice deficient in CLC7 show severe osteopetrosis. Although the number of osteoclasts in the bone tissue is normal, the osteoclasts fail to resorb the bone because they cannot acidify the extracellular resorption lacuna^47^. However, osteoclast-specific knockout Ano1 results in a significant reduction in osteoclast number. Although the expression of CLC7 and Ano1 is increased in the early stages of osteoclastogenesis, CLC7 resides in the late endosomal and lysosomal compartments, it is only highly expressed in the ruffled membrane and secretes Cl^−^ into the resorption lacuna in mature osteoclasts. Therefore, it can be inferred that the Cl^−^ current in preosteoclasts is mainly attributed to Ano1.

There are ~400 genes that encode ion channels, which is the second largest class of proven drug targets after G protein-coupled receptors (GPCRs). Ion channels play an important role in regulating ion homeostasis in many cells and are involved in a series of physiological processes^48,49^. An understanding of the expression and function of Ano1 in osteoclasts will allow the identification of a therapeutic target for osteoporosis or osteopetrosis patients. The structure of Ano1 has been resolved, and there are tremendous opportunities for identifying specific modulators for bone metabolic disease. The obvious regulatory effects of Ano1 on RANK during osteoclastogenesis and its antiresorptive effect make it an advantageous target. Screening Ano1-specific inhibitors for bone loss therapy in the future may be a promising approach. Considering Ano1 knockout mice cannot survive longer than 30 days^50^, the side-effect of these inhibitor need to be further explored. In the future, it will be better to use the osteoclast specific delivery strategy for the application of Ano1 inhibitor in osteoporosis treatment.

Taken together, our findings established Ano1 as a player in the field of osteoclast biology, advancing our understanding of osteoporosis and osteopetrosis. Ano1 overexpression promotes osteoclast formation and bone resorption, and osteoclast-specific Ano1 knockout reverses OVX induced bone loss by inhibiting osteoclastogenesis and function. The unique molecular mechanism of Ano1, including Cl^−^ channel activity and ability to physically interact with other proteins, contributes to its strong regulatory effects on osteoporosis and osteopetrosis and advances our understanding of bone metabolism related diseases.

## Methods

### Animals

To delete *Ano1* specifically in osteoclasts, the conditional KO mice were generated by crossing the *Ano1* floxed mice^19^(*Ano1*^*fl/fl*^, a generous gift from Dr. Min-Sheng Zhu) with the Ctsk-Cre transgenic mice (a generous gift from Dr. Weiguo Zou). We selected *Ctsk-Cre;Ano1*^*fl/fl*^ as experimental mice, *Ano1*^*fl/fl*^ littermates served as controls. Generation of osteoclast-specific *Ano1* transgenic mice, the plasmid (a generous gift from Dr. Sakamuri V. Reddy), which contains the *Acp5* promoter to drive osteoclast-specific gene expression, was used to generate *Acp5*-m*Ano1* transgenic mice (*Ano1 TG*). Mouse *Ano1* CDS fragment was amplified from mouse liver cDNA. *Acp5*-m*Ano1* plasmid was constructed by inserting the XhoI /NotI fragment of mAno1 into the corresponding sites of downstream of *Acp5* promoter. This plasmid was linearized with EcoRI, then the fragments of the *Acp5*-m*Ano1* were purified and microinjected into C57BL/6J mouse embryos and the embryos were then surgically transferred into pseudo-pregnant B6D2F1 (C57BL/6 X DBA2) female mice as foster mothers at the Laboratory Animal Research Center of Tsinghua University. Animals were bred and maintained under specific pathogen free (SPF) conditions in Animal Research Building of China Astronaut Research and Training Center (12-h light, 12-h dark cycles, temperature controlled at 21 ± 2 °C with free access to food and water). All the experimental procedures were approved by the Committees of Animal Ethics and Experimental Safety of the China Astronaut Research and Training Center (Reference number: ACC-IACUC-2020–004).

### Osteoclast differentiation

Mouse bone marrow-derived macrophages (BMMs) were obtained from cultures of bone marrow collected from tibias and femurs of 6–8-week-old mice. Bone marrow cells were flushed and collected with α-minimal essential media (α-MEM). Cells were cultured with complete α-MEM medium (α-MEM, 10% fetal bovine serum [FBS], and penicillin/streptomycin) in the presence of macrophage colony stimulating factor (M-CSF; 10 ng/ml, R&D Systems) for 1 day. Suspension cells were collected for osteoclast generation. Cells were cultured in induced medium-complete medium with 30 ng/ml M-CSF and 50 ng/ml receptor activator of nuclear factor kB ligand (RANKL; R&D Systems) for 5 days. The culture medium was replaced every 2 days.

### TRAP staining

TRAP staining was performed using an acid phosphatase kit according to the manufacturer’s instructions (Sigma-Aldrich, catalog no. 387). After 5 days of culture, RANKL-induced osteoclasts were fixed by 4% formaldehyde for 5 min at room temperature and rinsed thoroughly in deionized water. The plates were incubated in TRAP staining solution at 37 °C for 1 h protect from light. Following removal of the TRAP solution, the plates were washed three times with distilled water. TRAP-positive multinucleated cells containing three or more nuclei were counted under an inverted microscope (Nikon).

### Bone resorption assays

To analyze resorption pit formation, BMMs cells were seeded onto 24 wells Corning^®^ Osteo Assay Surface plate and culture with induced medium, After 5 days of culture, 100 µl of 10% bleach solution was added. Cells were incubated with the bleach solution for 5 min at room temperature. The wells were washed twice with distilled water and allowed to dry at room temperature for 3–5 h. Individual pits or multiple pit clusters were observed using microscope.

### Acridine orange staining

Acid production was determined using acridine orange as described previously. BMMs were induced by RANKL/M-CSF for 5 days and incubated in a-MEM containing 5 μg/ml of acridine orange (Sigma) for 15 min at 37 °C, washed, and chased for 10 min in fresh media. The cells were observed under a fluorescence microscope.

### Electrophysiology

Whole-cell patch clamp experiments were performed at room temperature (22–25 °C). The patch pipettes were made of borosilicate glass (Sutter Instruments, Novato, USA) and drawn using a P-97 puller (Sutter Instruments, Novato, USA) with a pipette resistance of 3–5 MΩ when immersed in the bath solution. All recordings were performed with an EPC10 amplifier controlled by Pulse software with a Digi LIH1600 interface (HEKA, Lambrecht, Germany). The data were low-pass filtered at 2.9 kHz and sampled at 10 kHz. The stimulation protocols consisted of voltage steps of 1150 ms duration from a holding potential of 0 mV, membrane voltage (*V*_m_) was clamped in steps of 20 mV from −80 to +160 mV, followed by −80 mV. The pipette solution used in whole-cell patch clamp recording contained CsCl 130 mM, EGTA 10 mM, MgATP 1 mM, MgCl_2_·6H_2_O 1 mM, and HEPES 10 mM. Standard CaCl_2_ (1 M, Sigma, USA) was added to produce various free Ca^2+^ concentrations; these were calculated using the CaEGTA Calculator V1.2, available online at http://www.stanford.edu/~cpatton/CaEGTA-NIST.htm. The pipette solution containing 600 nM free Ca^2+^ was prepared by addition of the standard CaCl_2_ solution to a final concentration of 8.69 mM and adjustment to pH 7.4 with CsOH. The bath solution for the whole-cell patch clamp recording contained NaCl 150 mM, MgCl_2_·6H_2_O 1 mM, HEPES 10 mM, glucose 10 mM, and mannitol 10 mM adjusted to pH 7.4 with NaOH. The osmotic pressure was in the range of 290–300 mOsm/L for pipette solution and 300–310 mOsm/L for bath solution, measured by an OM815 osmometer (Löser Messtechnik, Germany). HEPES solution: HEPES 20 mM, NaCl 128 mM, KCl 2.5 mM, CaCl_2_ 2.7 mM, MgCl_2_ 1 mM and glucose 16 mM, adjusted to pH 7.4 with NaOH. D-PBS solution: KCl 2.67 mM, KH_2_PO_4_ 1.47 mM, NaCl 138 mM, and Na_2_HPO_4_ 8.1 mM, adjusted to pH 7.4 with NaOH.

### Cell transfection

Cells for RNA interference were transfected with siRNA or NC using Lipofectamine RNAiMAX in OptiMEM as per the manufacturer’s instructions (Thermo Fisher Scientific).

Sequences of the siRNA sequence were as follows: *Ano1* siRNA, 5′-UUUAGACAAAAACCAAUAGAU-3′; *Clcn7* siRNA, 5′-UCCAAAAAUAUCAUUUCAGCA-3′; *Clcn4* siRNA, 5′-AUGUGUUAUAAUGUAUAGCUA-3′*CFTR* siRNA, 5′-AUCUUUCAAUCAAUACCACCC-3′; Negative control siRNA (NC): 5′-UUCUCCGAACGUGUCACGUTT-3′.

### RT-PCR and quantitative real-time PCR

Total RNA was extracted from cells or tissues using TRIzol reagent according to the manufacturer’s instructions. RNA was reverse transcribed into cDNA with the PrimeScript RT Reagent Kit (Takara, PR037A) according to the manufacturer’s instructions. Quantitative Real-Time PCR (QRT-PCR) using a TB Green™ Premix Ex Taq™ II (Takara, RR820A). Gapdh was used as normalization control for mRNA. mRNA primer sequences are listed in Supplementary Table 2. All primers were manufactured by BGI (Beijing, China).

### Western blotting

Cells were lysed in lysis buffer (RIPA buffer, 1 mM PMSF, phosphatase inhibitor Cocktail and protease inhibitor cocktail) on ice for 15 min. Bone tissues were ground with a mortar in liquid nitrogen and were lysed in lysis buffer at 4 °C for 30 min. Protein fractions were collected by centrifugation at 12,000×*g*, 4 °C for 10 min and then 10 mg of lysates were subjected to SDS–PAGE and transferred to nitrocellulose filter membrane (NC) membranes. The membranes were blocked with 5% skimmed milk and incubated with specific antibodies overnight. The antibody of used were listed: rabbit anti-Ano1 (1:1000, abclonal, Cat. No. A10498, polyclonal), rabbit anti-p-Syk antibody (1:1000, abclonal, Cat. No. AP0501, polyclonal), rabbit anti-Syk antibody (1:1000, abclonal, Cat. No. A2123, polyclonal), rabbit anti-Clcn4 antibody (1:1000, abclonal, CatNo.A13790, polyclonal), rabbit anti-Clcn7 antibody (1:1000, abclonal, Cat. No. A6886, polyclonal), rabbit anti-CFTR antibody (1:1000, abclonal, Cat. No. A8386, polyclonal), rabbit anti-p-Akt antibody (1:1000, Cell Signaling Technology, Cat. No. 4060, polyclonal), rabbit anti-Akt antibody (1:1000, Cell Signaling Technology, Cat. No. 2920, polyclonal), rabbit anti-p-CaMKIV antibody (1:1000, ImmunoWay, Cat. No. YP0043, polyclonal), rabbit anti-CaMKIV antibody (1:1000, Cell Signaling Technology, Cat. No. 4032, polyclonal), rabbit anti-Creb antibody (1:1000, Cell Signaling Technology, Cat. No. 9197, Monoclonal), rabbit anti-p-Creb antibody (1:1000, Cell Signaling Technology, Cat. No. 9198, Monoclonal), rabbit anti-Plcγ2 antibody (1:1000, Cell Signaling Technology, Cat. No. 55512, Monoclonal), rabbit anti-p-Plcγ2 antibody (1:1000, Cell Signaling Technology, Cat. No. 3871, polyclonal), rabbit anti-Btk antibody (1:1000, Cell Signaling Technology, Cat. No. 5082, polyclonal), rabbit anti-Btk antibody (1:1000, Proteintech, Cat. No. 21581-1-AP, polyclonal), rabbit anti-TRAF6 antibody (1:200, Abcam, CatNo.ab137452, polyclonal), mouse anti-RANK antibody (1:200, Abcam, Cat. No. ab13918, polyclonal), rabbit anti-Gapdh antibody (1:5000, Abways, Cat. No. AB0036, Monoclonal).

### Coimmunoprecipitation

Mouse bone marrow-derived macrophages (BMMs) were obtained from cultures of bone marrow collected from tibias and femurs of 6–8-week-old mice. BMMs were flushed and collected with α-minimal essential media (α-MEM). Cells were cultured with complete α-MEM medium in the presence of M-CSF (10 ng/ml, R&D Systems) for 1 day. Suspension cells were collected and seeded on 10 cm dishes for osteoclast induction. Cells were cultured in induced medium-complete medium with 30 ng/ml M-CSF and 50 ng/ml RANKL; R&D Systems for 5 days. Control cells only treated with 30 ng/ml M-CSF for 5 days. Osteoclasts were transfected with Ano1 siRNA, Ano1 plasmid, mutation of Ano1 plasmid and treated with Benzbromarone on day 3 and collected samples on day 5 after RANKL induction. Cells were lysed in RIPA lysis solution (RIPA buffer, protease inhibitor, PMSF) and total protein was incubated with anti-RANK or Ano1 antibody for 2 h at 4 °C. Antibodies were precipitated using ProteinA/G agarose. The agarose was washed 4 more times and dissolved in 2× Laemmli buffer. The equal amount of samples was subjected to immunoblot analysis by using specific antibodies against RANK, Ano1, or TRAF6.

### Immunofluorescence

BMMs were treated with or without RANKL. After 5 days, fixed cells were incubated with anti-Ano1 and anti-RANK antibodies (1:500) overnight, followed by incubation of FITC or TRITC conjugated second antibodies (1:200) for 45 min. Nuclei were stained with Hoechst (blue) followed by confocal fluorescent microscopy.

### Micro-computed tomography (Micro-CT) analysis

The mouse femurs were skinned and fixed in 70% ethanol. For the distal femur, the whole secondary spongiosa of the left distal femur from each mouse was scanned ex vivo using a microCT system (mCT40, SCANCO MEDICAL, Switzerland). Briefly, 640 slices with a voxel size of 10 mm were scanned in the distal femur, and 80 slices of trabecular bone proximal to the distal growth plate was selected for analyzing the bone volume per tissue volume (BV/TV), trabecular number (Tb.N), trabecular thickness (Tb.Th) and trabecular spacing (Tb.Sp), and the structure model index (SMI).

### Histological analysis

The mouse tibias were skinned and fixed in 4% paraformaldehyde for 48 h. Tissues were embedded with paraffin after decalcification with 10% EDTA for 10–15 days, and 5–7 μm sections were prepared on a rotation microtome. The sections were incubated with TRAP staining solution at 37 °C for 1 h protect from light. Methyl green or hematoxylin was used for counterstaining. Images were acquired with microscope and statistical analyses were performed with the BioquantOsteo Analysis System.

### Serum analysis

The analyses were performed according to the manufacturer’s instructions for serum concentrations of CTX-1 (ELISA, Sangon Biotech).

### Intracellular Cl^−^ concentration measurements

Osteoclasts were seeded onto gelatin-coated 35-mm glass bottom dishes. On the day of imaging, cells were washed with PBS and incubated with 5 mM N-(ethoxycarbonylmethyl)-6-methoxyquinolinium bromide (MQAE, molecular probes) for 30 min at room temperature in normal Tyrode’s solution (150 mM Cl^−^) or low Cl^−^ Tyrode’s solution with 5% serum. Tyrode solution is composed of: 140 mM NaCl, 5 mM KCl, 2 mM CaCl_2_, 1 mM MgCl_2_, 10 mM glucose, and 10 mM HEPES, pH 7.2. Low Cl^−^ solution: 5 mM Cl^−^ and 145 mM gluconate. Fluorescence images were performed using a confocal laser scanning microscopy (CLSM, Leica SP5, Germany) with excitation light 350 nm and emission light 460 nm. Changes in [Cl^−^]_i_ were represented by the changes in the fluorescence intensity. The average fluorescence intensity of each cell was analyzed by Image J software.

### Intracellular Ca^2+^ measurement

Osteoclasts derived from *Ano1*^*fl/fl*^, *Ctsk-Cre;Ano1*^*fl/fl*^, *WT*, and *Ano1 TG* mice were seeded on confocal dish and induced by RANKL for 5 days to measure intracellular Ca^2+^^51^. The cells were loaded with 5 μM fluo-4, AM (Molecular Probe) for 20 min at 37 °C in Tyrode solution, then rinsed twice with Tyrode solution and mounted on the inverted stage of a confocal scope. Fluorescence excitation was performed using 488 nm laser, and detection filters were set at 530 nm. Images were acquired every 3 s and analyzed using Interactive Data Language (IDL, Research Systems) software. Cells were scanned for 20–30 s to obtain *F*_resting_ (*F*), then replaced the solution with 0 Ca^2+^ Tyrode solution including 4 mM EGTA (Invitrogen), 5 μM thapsigargin (Molecular probes), and 10 μM A23187 (Sigma). Stored calcium was released to the cytoplasm immediately. We defined the peak value as F_ER release_. Added 100 μM BAPTA, AM into solution to obtain *F*_min_. Then replaced the solution with 10 mM Ca^2+^, 5 μM thapsigargin, 12 μM A23187 (Sigma), 3 μM FCCP (Sigma), and 20 mM 2-DG (Sigma) in Tyrode solution. The stable value was *F*_max_. Finally, [Ca^2+^]_i_ was calibrated using the equation [Ca^2+^] = *K*_d_×(*F*–*F*_min_)/(*F*_max_–*F*).

### Microarray analysis

For transcriptomic analysis, osteoclasts derived from 3 *Ctsk-Cre;Ano1*^*fl/fl*^ mice and 3 their littermate controls *Ano1*^*fl/fl*^ mice were induced by RANKL for 5 days. These osteoclasts were collected to extract RNA. After the quality control of the sample, the quantile algorithm is used to standardize the data. The transcriptomic analysis was performed by oebiotech (Shanghai, China). Agilent SurePrint G3 Mouse Gene Expression v2 8x60K Microarray (Design ID: 074809) chip was used in this project. We have deposited microarray datasets in the Gene Expression Omnibus (GEO) repository, the accession codes is GSE193800 and associated hyperlinks is https://www.ncbi.nlm.nih.gov/geo/query/acc.cgi?acc=GSE193800.

### Preparation of human bone tissue

The bone tissues of 17 non-osteoporotic people and 15 osteoporotic patients with fracture at between 60 and 80 years of age from Peking University the Third Hospital. Non-osteoporotic human and osteoporotic patients who had fracture caused by falling without obvious violence were included in our study. Patients who subjected to diabetes, malignancy, hyperparathyroidism and other sever bone diseases were excluded from our study. We also excluded the patients who had taken glucocorticoids, estrogen, or anti-osteoporosis drugs within 1 year. We obtained all participants informed consents. The classification of the patients into the osteoporotic and non-osteoporotic groups was based on DXA evaluation. We measured the *T*-score for BMD in the spine of women. A *T*-score of −2.5 or lower qualifies as osteoporosis. Others were control patients (*T* > −2.5). We obtained informed consent from all participants. The study protocol conformed to the ethical guidelines of the 1975 Declaration of Helsinki and all the clinical procedures were approved by Peking University the Third Hospital (Beijing, China) (Reference number: IRB00006761-M2021216).

### OVX mouse model

All the female mice used were maintained under standard animal housing conditions. The 3-month female mice were ovariectomy (OVX) or sham-operated at 3 months of age. At 3 months after surgery (6 months of age), serum and bone tissues from sham-operated and OVX mice were collected. All of the experimental procedures were approved by the Committees of Animal Ethics and Experimental Safety of the China Astronaut Research and Training Center.

### Statistical analysis

Cell-based experiments were performed at least three independent replicates. Animals were randomized into different groups and at least 3 mice were used for each group, unless otherwise stated. The data are presented as mean ± s.e.m. Student’s *t*-test was used for statistical evaluations of two group comparisons. Statistical analysis with more than two groups was performed with one-way analysis of variance (ANOVA). All statistical analyses were performed with Prism software (Graphpad prism for windows, version 9.0).

### Reporting summary

Further information on research design is available in the Nature Research Reporting Summary linked to this article.

## Supplementary information

Supplementary information

Reporting Summary

## Data Availability

The microarray datasets that support the findings of this study have been deposited in the Gene Expression Omnibus (GEO) repository, the accession codes is GSE193800. The source data relevant to Figs. 1–6 and Supplementary Figs. 1–5 are provided as a Source Data file. Source data are provided with this paper.

## Source data

Source data
