# Supplementary material for: Anoctamin 1 controls bone resorption by coupling Cl− channel activation with RANKL-RANK signaling transduction
Source: Nat Commun. 2022 May 24;13:2899. doi: 10.1038/s41467-022-30625-9 (PMC9130328; doi:10.1038/s41467-022-30625-9)

Anoctamin 1 controls bone resorption by coupling Cl^- channel activation with RANKL-RANK signaling transduction

Weijia Sun[1, 6], Shuai Guo[2, 3, 6], Yuheng Li[1, 4, 6], JianWei Li[1], Caizhi Liu[1], Yafei Chen[2], Xuzhao Wang[2], Yingjun Tan[1], Hua Tian[5], Cheng Wang[5], Ruikai Du[1], Guohui Zhong[1], Sai Shi[2], Biao Ma[2], Chang Qu[2], Jingxuan Fu[2], Xiaoyan Jin[1], Dingsheng Zhao[1], Yong Zhan[2], Shukuan Ling[1*], Hailong An[2*], and Yingxian Li[1*]

[1]State Key Laboratory of Space Medicine Fundamentals and Application, China Astronaut Research and Training Center, Beijing, 100094, China; [2]Key Laboratory of Molecular Biophysics, Hebei Province, Institute of Biophysics, School of Sciences, Hebei University of Technology, Tianjin, 300401, China; [3]School of Life Science, Hebei University, Baoding, Hebei, 071002, China; [4]The Key Laboratory of Aerospace Medicine, Ministry of Education, The Fourth Military Medical University, Xi'an, Shaanxi, China; [5]Department of Orthopedics, Peking University the Third Hospital, Beijing, 100083, China.

[6]These authors contributed equally: Weijia Sun, Shuai Guo and Yuheng Li.

[*]Correspondence should be addressed to Yingxian Li (email: yingxianli@aliyun.com), Hailong An (email: hailong_an@hebut.edu.cn) or Shukuan Ling (email: sh2ling@126.com)

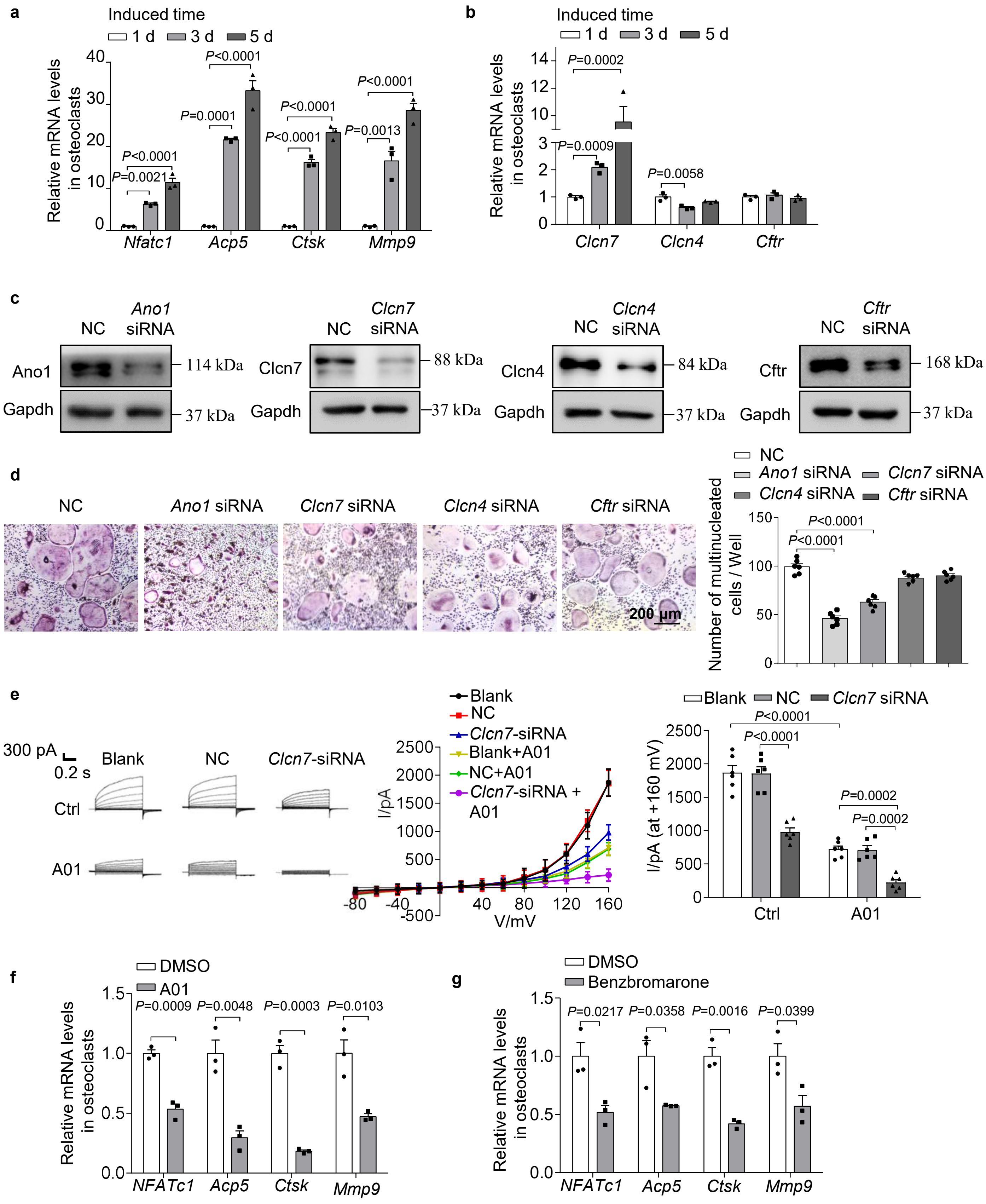
a
Induced time
1 d
3 d
5 d
Relative mRNA levels in osteoclasts
40
30
20
10
0
P<0.0001
P=0.0021
P=0.0001
P<0.0001
P<0.0001
P<0.0001
P=0.0013
P<0.0001
Nfatc1
Acp5
Ctsk
Mmp9
b
Induced time
1 d
3 d
5 d
Relative mRNA levels in osteoclasts
14
12
10
3
2
1
0
P=0.0002
P=0.0009
P=0.0058
Clcn7
Clcn4
Cftr
c
NC
Ano1 siRNA
Ano1
Gapdh
114 kDa
37 kDa
NC
Clcn7 siRNA
Clcn7
Gapdh
88 kDa
37 kDa
NC
Clcn4 siRNA
Clcn4
Gapdh
84 kDa
37 kDa
NC
Cftr siRNA
Cftr
Gapdh
168 kDa
37 kDa
d
NC
Ano1 siRNA
Clcn7 siRNA
Clcn4 siRNA
Cftr siRNA
200 μm
NC
Ano1 siRNA
Clcn7 siRNA
Clcn4 siRNA
Cftr siRNA
Number of multinucleated cells / Well
150
100
50
0
P<0.0001
P<0.0001
e
300 pA
0.2 s
Blank
NC
Clcn7-siRNA
Ctrl
A01
Blank
NC
Clcn7-siRNA
Blank+A01
NC+A01
Clcn7-siRNA + A01
I/pA
2500
2000
1500
1000
500
-500
-80
-40
40
80
120
160
V/mV
Blank
NC
Clcn7 siRNA
I/pA (at +160 mV)
2500
2000
1500
1000
500
0
P<0.0001
P<0.0001
P=0.0002
P=0.0002
Ctrl
A01
f
DMSO
A01
Relative mRNA levels in osteoclasts
1.5
1.0
0.5
0.0
P=0.0009
P=0.0048
P=0.0003
P=0.0103
NFATc1
Acp5
Ctsk
Mmp9
g
DMSO
Benzbromarone
Relative mRNA levels in osteoclasts
1.5
1.0
0.5
0.0
P=0.0217
P=0.0358
P=0.0016
P=0.0399
NFATc1
Acp5
Ctsk
Mmp9

Supplementary Fig. 1 Screening of chloride channels in osteoclast.

(**a**) QRT-PCR analysis of *NFATc1*, *Acp5*, *Ctsk* and *Mmp9* mRNA levels during osteoclast differentiation (n = 3). (**b**) QRT-PCR analysis of *Clcn7*, *Clcn4* and *Cftr* mRNA levels during osteoclast differentiation (n = 3). (**c**) Western blot analysis of Ano1, Clcn7, Clcn4, Cftr protein levels in RANKL-induced osteoclasts after treatment with siRNA or NC for 5 days. (**d**) Representative images of TRAP staining in RANKL-induced osteoclasts after treatment with siRNA or NC for 5 days (left). Scale bar, 200 μm. Quantification of the number of multinucleated cells per well (right) (n = 6). (**e**) Whole-cell recording of chloride currents in osteoclasts. *Clcn7* siRNA or its negative control (NC) transfected osteoclasts were treated with 20 μM CaCCinh-A01 (A01) or its control. Blank, without any treatment (n = 6). (**f**) QRT-PCR analysis of *NFATc1*, *Acp5*, *Ctsk* and *Mmp9* mRNA levels in osteoclasts after treatment with A01 (20 μM) for 5 days (n = 3). (**g**) QRT-PCR analysis of *NFATc1*, *Acp5*, *Ctsk* and *Mmp9* mRNA levels in osteoclasts after treatment with Ano1 inhibitor Benzbromarone (10 μM) for 5 days (n = 3). All data are the mean ± s.e.m. (a and b) Statistical analysis with more than two groups was performed with one-way analysis of variance (ANOVA) with Tukey's multiple comparisons test to determine group differences; (d, f and g) Two-tailed unpaired Student's t-test was used for statistical evaluations of two group comparisons; (e) Statistical analysis with more than two groups was performed with two-way analysis of variance (ANOVA) with Šídák post-hoc test to determine group differences. Source data are provided as a Source Data file.

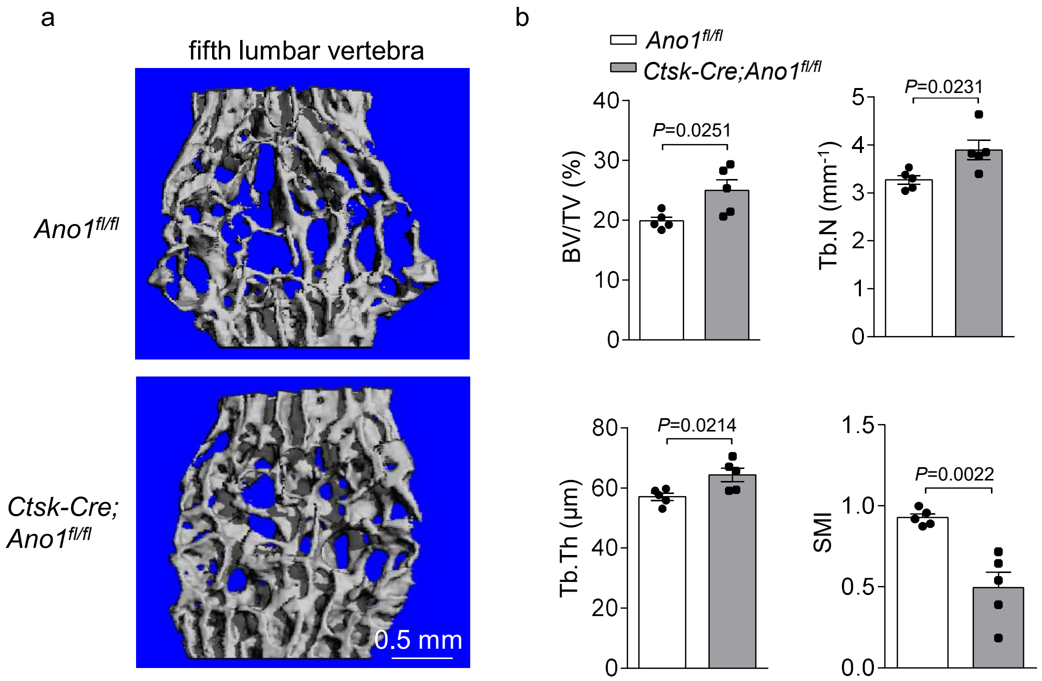

Supplementary Fig. 2 The vertebral phenotype of osteoclast-specific *Ano1* knockout mice

(**a**) Representative images showing three-dimensional trabecular architecture by micro-CT reconstruction at the fifth lumbar vertebra from *Ano1*$^{fl/fl}$ and *Ctsk-Cre;Ano1*$^{fl/fl}$ male mice at 2 months old. Scale bar, 0.5 mm. (**b**) Micro-CT measurements for BV/TV, Tb.N, Tb.Th, and SMI at the fifth lumbar vertebra of mice (n = 5). BV/TV, ratio of bone volume to tissue volume; Tb.N, trabecular number; Tb.Th, trabecular thickness; SMI, structure model index. All data are the mean ± s.e.m. Statistical analysis for comparison of two groups was performed using two-tailed unpaired Student's t-test. Source data are provided as a Source Data file.

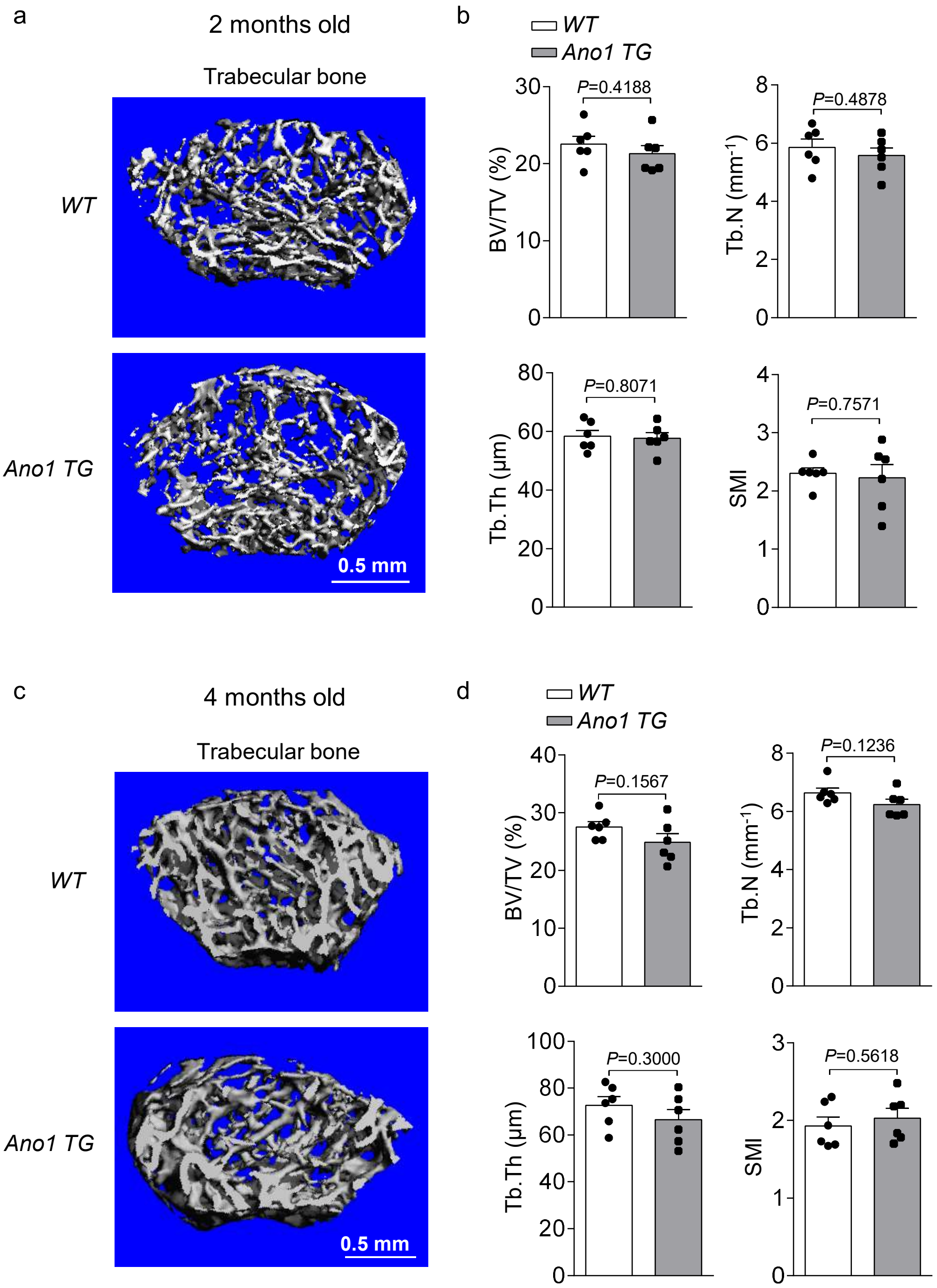
a
2 months old
Trabecular bone
WT
Ano1 TG
0.5 mm
b
WT
Ano1 TG
BV/TV (%)
P=0.4188
Tb.N (mm-1)
P=0.4878
Tb.Th (μm)
P=0.8071
SMI
P=0.7571
c
4 months old
Trabecular bone
WT
Ano1 TG
0.5 mm
d
WT
Ano1 TG
BV/TV (%)
P=0.1567
Tb.N (mm-1)
P=0.1236
Tb.Th (μm)
P=0.3000
SMI
P=0.5618

Supplementary Fig. 3 The phenotype of osteoclast-specific *Ano1* transgenic mice at 2 months and 4 months of age

(**a**) Representative images showing three-dimensional trabecular architecture by micro-CT reconstruction at the distal femurs from *WT* and *Ano1 TG* mice at 2 months old. Scale bar, 0.5 mm. (**b**) Micro-CT measurements for BV/TV, Tb.N, Tb.Th, Tb.Sp, and SMI at the distal femurs from *WT* (n = 6) and *Ano1 TG* mice (n = 6) at 2 months old. (**c**) Representative images showing three-dimensional trabecular architecture by micro-CT reconstruction at the distal femurs from *WT* and *Ano1 TG* mice at 4 months old. Scale bar, 0.5 mm. (**d**) Micro-CT measurements for BV/TV, Tb.N, Tb.Th, Tb.Sp, and SMI at the distal femurs from *WT* (n = 6) and *Ano1 TG* mice (n = 6) at 4 months old. All data are the mean ± s.e.m. Statistical analysis for comparison of two groups was performed using two-tailed unpaired Student's t-test. Source data are provided as a Source Data file.

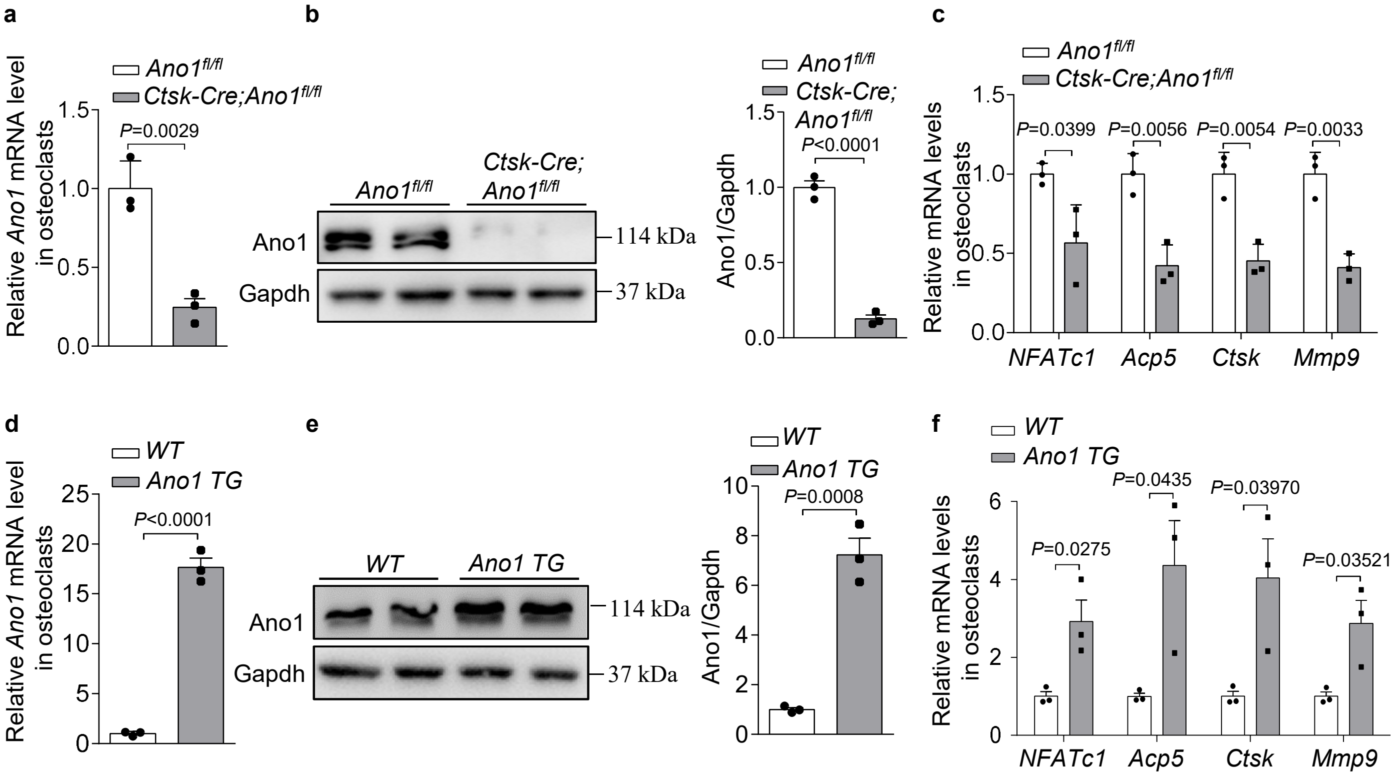

Supplementary Fig. 4 ***Ano1*** **knockout inhibit osteoclasts function** ***and Ano1*** **overexpression enhances osteoclast function.**

(**a** and **b**) QRT-PCR analysis of *Ano1* mRNA level and western blot analysis of Ano1 protein level in RANKL-induced osteoclasts isolated from *Ano1*$^{fl/fl}$ and *Ctsk-Cre;Ano1*$^{fl/fl}$ mice for 5 days (n = 3). (**c**) QRT-PCR analysis of *NFATc1*, *Acp5*, *Ctsk* and *Mmp9* mRNA levels in RANKL-induced osteoclasts isolated from *Ano1*$^{fl/fl}$ and *Ctsk-Cre;Ano1*$^{fl/fl}$ mice for 5 days (n = 3). (**d** and **e**) QRT-PCR analysis of *Ano1* mRNA level and western blot analysis of Ano1 protein level in RANKL-induced osteoclasts isolated from WT and *Ano1 TG* mice for 5 days (n = 3). (**f**) QRT-PCR analysis of *NFATc1*, *Acp5*, *Ctsk* and *Mmp9* mRNA levels in RANKL-induced osteoclasts isolated from WT and *Ano1 TG* mice for 5 days (n = 3). All data are the mean ± s.e.m. Two-tailed unpaired Student's t-test was used for statistical evaluations of two group comparisons. Source data are provided as a Source Data file.

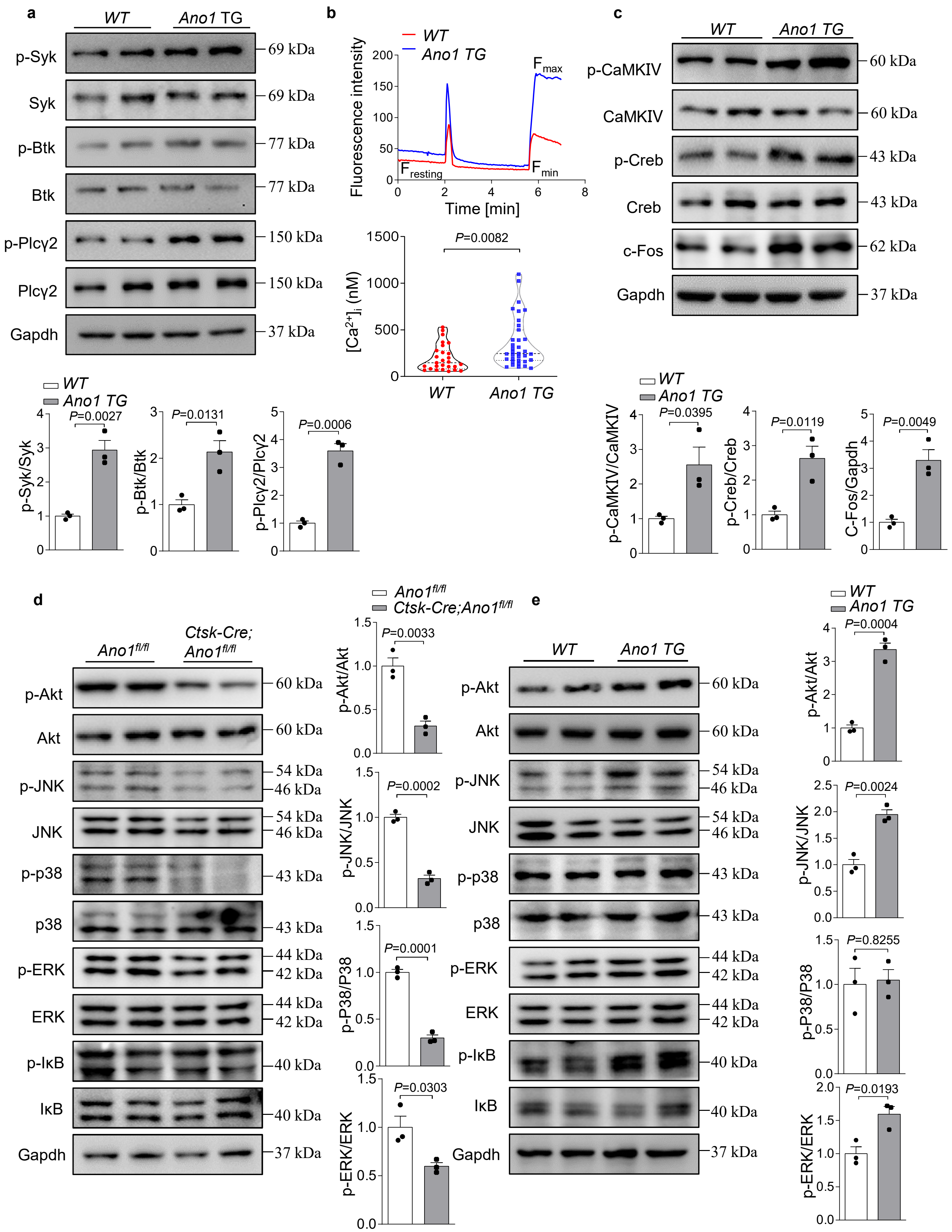

a
WT
Ano1 TG
p-Syk
Syk
p-Btk
Btk
p-Plcγ2
Plcγ2
Gapdh
69 kDa
69 kDa
77 kDa
77 kDa
150 kDa
150 kDa
37 kDa
WT
Ano1 TG
p-Syk/Syk
P=0.0027
p-Btk/Btk
P=0.0131
p-Plcγ2/Plcγ2
P=0.0006
b
WT
Ano1 TG
Fluorescence intensity
F max
F resting
F min
Time [min]
[Ca2+]i (nM)
P=0.0082
WT
Ano1 TG
c
WT
Ano1 TG
p-CaMKIV
CaMKIV
p-Creb
Creb
c-Fos
Gapdh
60 kDa
60 kDa
43 kDa
43 kDa
62 kDa
37 kDa
WT
Ano1 TG
p-CaMKIV/CaMKIV
P=0.0395
p-Creb/Creb
P=0.0119
C-Fos/Gapdh
P=0.0049
d
Ano1fl/fl
Ctsk-Cre; Ano1fl/fl
p-Akt
Akt
p-JNK
JNK
p-p38
p38
p-ERK
ERK
p-IκB
IκB
Gapdh
60 kDa
60 kDa
54 kDa
46 kDa
54 kDa
46 kDa
43 kDa
43 kDa
44 kDa
42 kDa
44 kDa
42 kDa
40 kDa
40 kDa
37 kDa
Ano1fl/fl
Ctsk-Cre;Ano1fl/fl
p-Akt/Akt
P=0.0033
p-JNK/JNK
P=0.0002
p-P38/P38
P=0.0001
p-ERK/ERK
P=0.0303
e
WT
Ano1 TG
p-Akt
Akt
p-JNK
JNK
p-p38
p38
p-ERK
ERK
p-IκB
IκB
Gapdh
60 kDa
60 kDa
54 kDa
46 kDa
54 kDa
46 kDa
43 kDa
43 kDa
44 kDa
42 kDa
44 kDa
42 kDa
40 kDa
40 kDa
37 kDa
WT
Ano1 TG
p-Akt/Akt
P=0.0004
p-JNK/JNK
P=0.0024
p-P38/P38
P=0.8255
p-ERK/ERK
P=0.0193

Supplementary Fig. 5 The function of Ano1 in osteoclast is dependent on both its channel activity and regulation on RANKL-RANK signaling.

(**a**) Western blot analysis of the phosphorylation levels of Syk, Btk, and Plcγ2 in *WT* and *Ano1 TG* osteoclasts (top). The quantification of the phosphorylation levels of Syk, Btk, and Plcγ2 in osteoclasts (below) (n = 3). (**b**) Resting $[Ca2+]_i$ in osteoclasts from *WT* mice and *Ano1 TG* mice, n = 27 (*WT*) and n = 37 (*Ano1 TG*) cells pooled from three independent experiments. (**c**) Western blot analysis of p-CaMKIV, p-Creb, and c-Fos protein levels in *WT* and *Ano1 TG* osteoclasts (top). The quantification of p-CaMKIV, p-Creb, and c-Fos protein levels in osteoclasts (below) (n = 3). (**d**) Western blot analysis of the phosphorylation levels of Akt, JNK, p38, ERK, and IκB in $Ano1^{fl/fl}$ and $Ctsk\text{-}Cre;Ano1^{fl/fl}$ osteoclasts (left). The quantification of the phosphorylation levels of Akt, JNK, p38, ERK, and IκB in osteoclasts (right) (n = 3). (**e**) Western blot analysis of the phosphorylation levels of Akt, JNK, p38, ERK, and IκB in *WT* and *Ano1 TG* osteoclasts (left). The quantification of the phosphorylation levels of Akt, JNK, p38, ERK, and IκB in osteoclasts (right) (n = 3). All data are the mean ± s.e.m. Two-tailed unpaired Student's t-test was used for statistical evaluations of two group comparisons. Source data are provided as a Source Data file.

Supplementary Table 1. Clinical features of fracture patients involved in bone specimens analysis

**Group**	**Age**	**Gender**	**T score for BMD at Spine**	**Diagnosis**	**Numbers**
Non-osteoporosis	60~74	Woman	T > -2.5	Femoral neck fracture	n=17
Osteoporosis	60~79	woman	T≤-2.5	Femoral neck fracture	n=15

Supplementary Table 2. mRNA Primers are used as follows

Mouse primers

Gapdh-Forward	5’- AACATCAAATGGGGTGAGGCC-3’
Gapdh-Reverse	5’- GTTGTCATGGATGACCTTGGC-3’
NFATc1-Forward	5’- ACGCTACAGCTGTTCATTGG -3’
NFATc1-Reverse	5’- CTTTGGTGTTGGACAGGATG -3
Acp5-Forward	5’- GCGACCATTGTTAGCCACATACG -3’
Acp5-Reverse	5’- CGTTGATGTCGCACAGAGGGAT -3’
Ctsk-Forward	5’- GCGTTGTTCTTATTCCGAGC -3’
Ctsk-Reverse	5’- CAGCAGAGGTGTGTACTATG -3’
Mmp9-Forward	5’- GCTGACTACGATAAGGACGGCA -3’
Mmp9-Reverse	5’- GCGGCCCTCAAAGATGAACGG -3’
ANO1-Forward	5’- CCCGTGCCAGTCACCTTTTT -3’
ANO1-Reverse	5’- TCATCTGCTTCCGTTTCCAGT -3’
ANO2-Forward	5’- TTCCAGCCTACACCCTAGCC -3’
ANO2-Reverse	5’- TCCTCTGGTTGTCGTGAAAGT -3’
Clcn1-Forward	5’- GGGACGTGTGCTGAGAAGG -3’
Clcn1-Reverse	5’- GGGCATAGGTCCACTTGTAGG -3’
Clcn2-Forward	5’- GCGATGGCATAGTCCATAGCC -3’
Clcn2-Reverse	5’- CCAGATGTCGCATTTGTTCTGT -3’
Clcn3-Forward	5’- GACTGGGTGCGAGAGAAGTG -3’
Clcn3-Reverse	5’- CATCCTGACCAGGCGTCATAC -3’
Clcn4-Forward	5’- TCTCCCTGGTACGTGTGTTC -3’
Clcn4-Reverse	5’- ACGAGGGTGACAGTCTTGATTAG -3’
Clcn5-Forward	5’- GGAGCCAATCCCTGGTGTAG -3’
Clcn5-Reverse	5’- CTCTCGGTGCCTATCTCGGT -3’
Clcn6-Forward	5’- GCTGGTGGGTCTCTTTGTGG -3’
Clcn6-Reverse	5’- CTGGCTCTATCAAGACAAGAAGG -3’
Clcn7-Forward	5’- ACACAGCGTCTAATCACAAC -3’
Clcn7-Reverse	5’- GTCCTTCAGCCTCAGTCG -3’
CFTR-Forward	5’- CATCGCGGTAACCGTCCT -3’
CFTR-Reverse	5’- CCGCAGTTTTACTCCGCAG -3’

Human primers

GAPDH-Forward	5’- ACAACTTTGGTATCGTGGAAGG-3’
GAPDH-Reverse	5’- GCCATCACGCCACAGTTTC-3’
ANO1-Forward	5’- ACTACCACGAGGATGACAAGC -3’
ANO1-Reverse	5’- TCTCTGCACAGCACGTTCC -3’
NFATc1-Forward	5’- CCTGTCCCCTACGTCCTACA -3’
NFATc1-Reverse	5’- GGCGCACAAGGAAAAGTCTG -3’
CTSK-Forward	5’-AAGCCAGACAACAGATTTCCAT-3’
CTSK-Reverse	5’-GGATCATTTGAAGCACAAACAA-3’
ACP5-Forward	5’-GGAGGGAATAAAGGCTCAGG-3’
ACP5-Forward	5’-GGAACTCAGCAAAGGTGAGC-3’
MMP9-Forward	5’-CGTCGGTCCGTCCGCTA-3’
MMP9-Reverse	5’-TCAGCCCTCACCTCGGTACT-3’

The sequence of primers were used for detecting mRNA expression by quantitative PCR.

Uncropped versions of blots

Supplementary Fig. 1

c

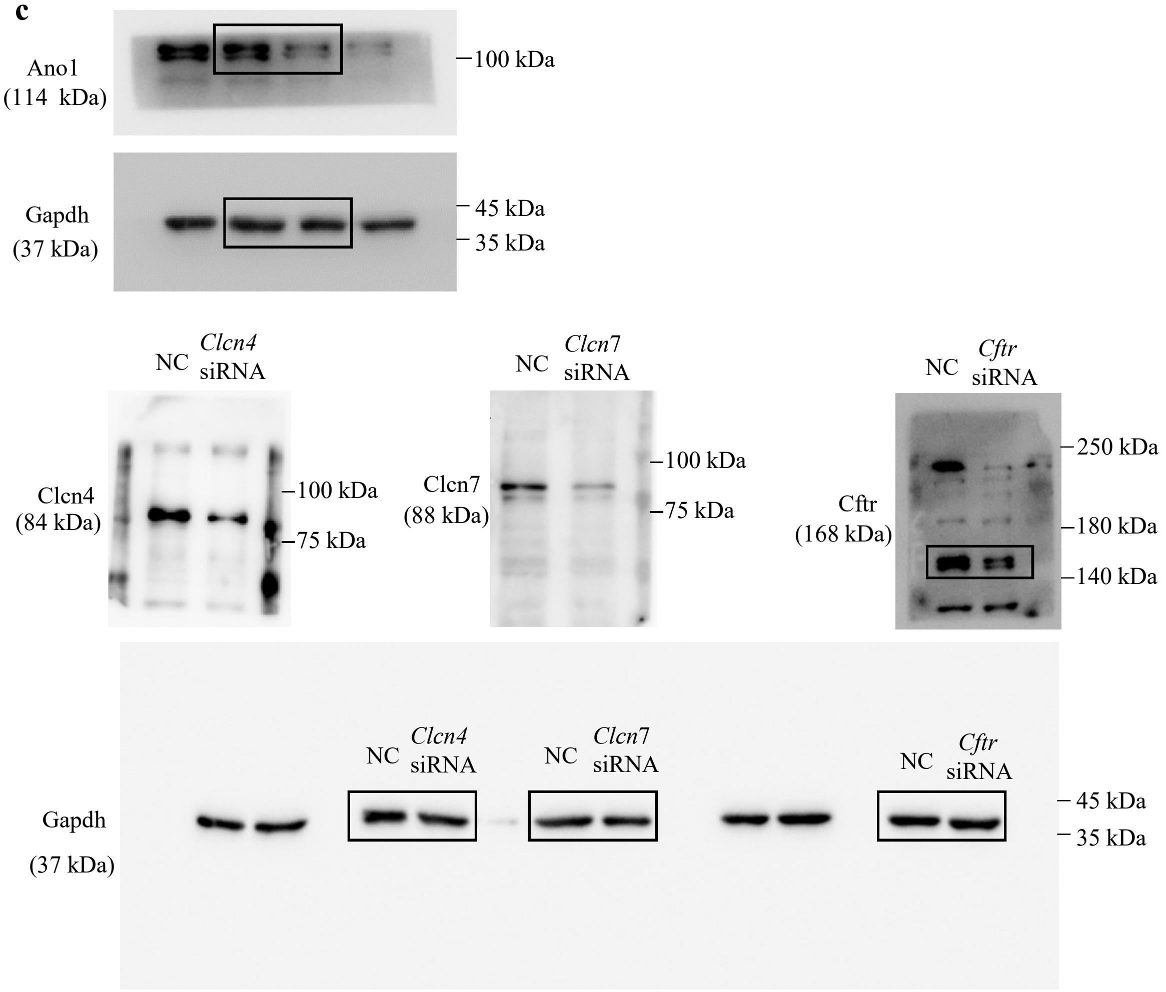

Supplementary Fig. 4

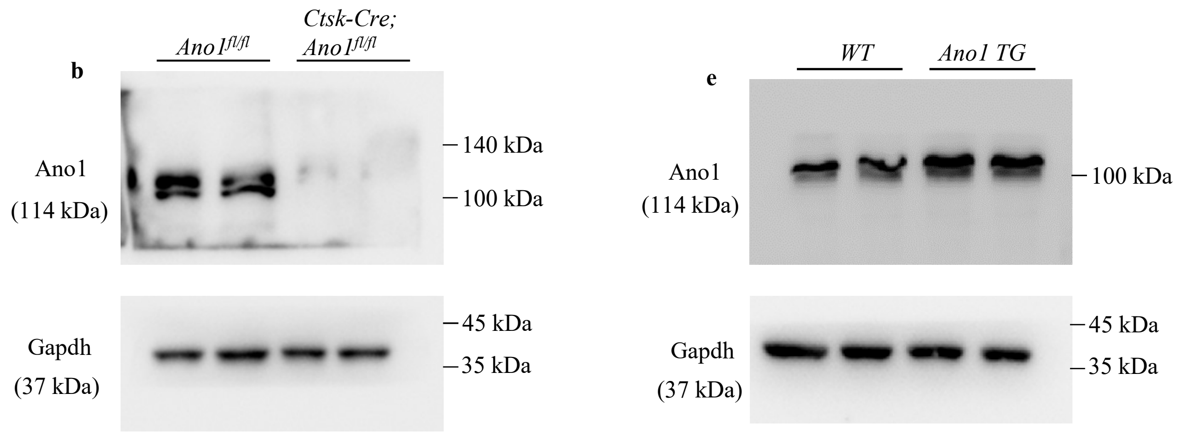

Supplementary Fig. 5

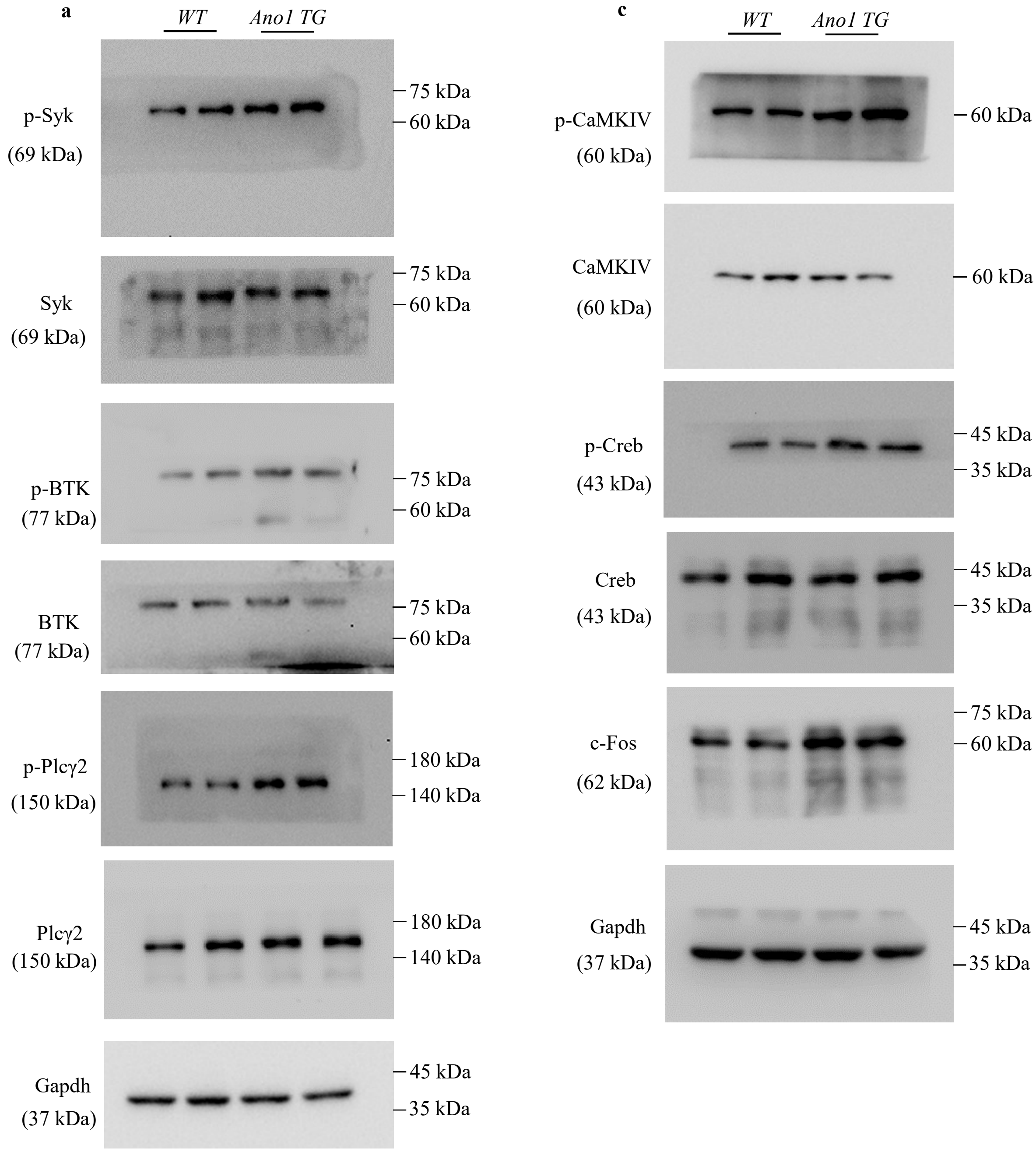

Supplementary Fig. 5

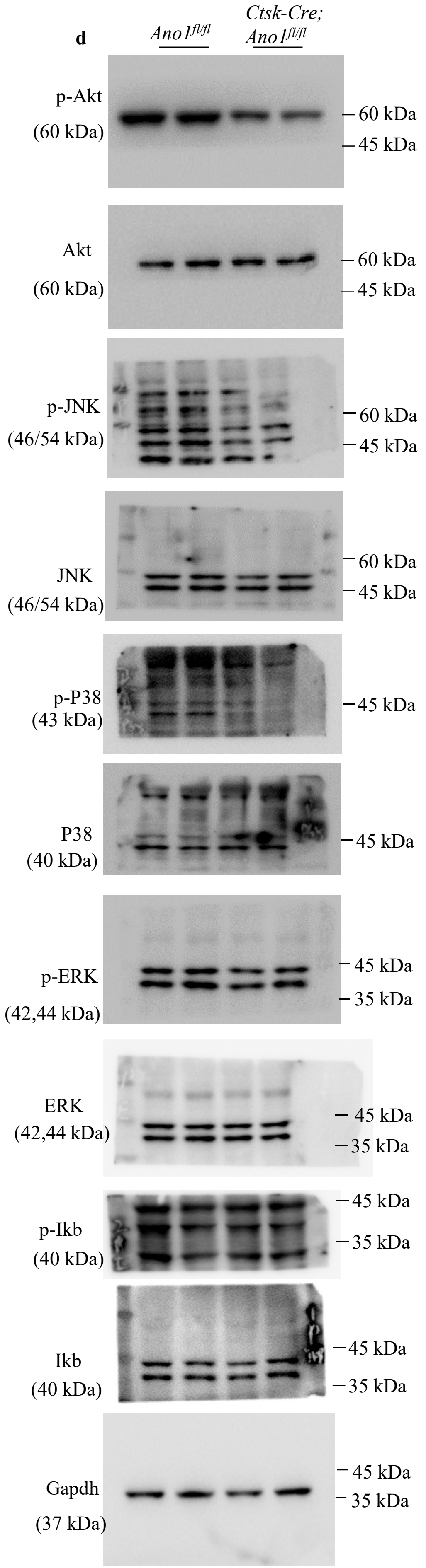

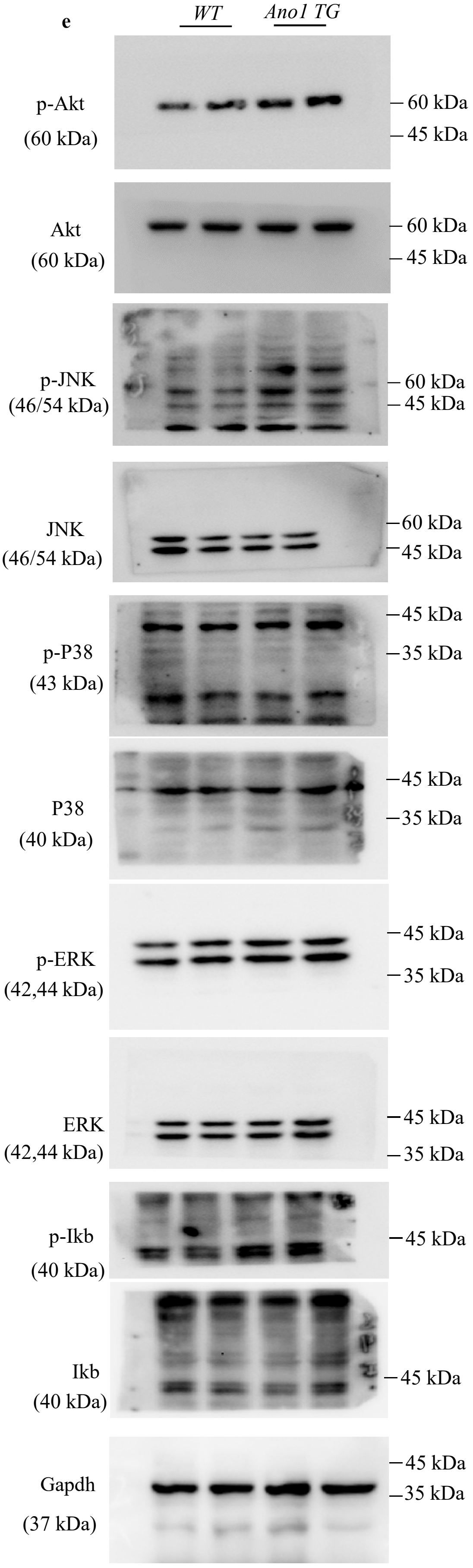

Supplement: Supplementary file 1 — Supplementary information [file 41467_2022_30625_MOESM1_ESM.pdf]
