# Supplementary material for: Anoctamin 1 controls bone resorption by coupling Cl− channel activation with RANKL-RANK signaling transduction
Source: Nat Commun. 2022 May 24;13:2899. doi: 10.1038/s41467-022-30625-9 (PMC9130328; doi:10.1038/s41467-022-30625-9)
Supplement: Supplementary file 2 — Reporting Summary [file 41467_2022_30625_MOESM2_ESM.pdf]

nature portfolio

Corresponding author(s): Yingxian Li

Last updated by author(s): Mar 28, 2022

Reporting Summary

Nature Portfolio wishes to improve the reproducibility of the work that we publish. This form provides structure for consistency and transparency in reporting. For further information on Nature Portfolio policies, see our Editorial Policies and the Editorial Policy Checklist.

Statistics

For all statistical analyses, confirm that the following items are present in the figure legend, table legend, main text, or Methods section.

n/a	Confirmed	
☐	☒	The exact sample size (*n*) for each experimental group/condition, given as a discrete number and unit of measurement
☐	☒	A statement on whether measurements were taken from distinct samples or whether the same sample was measured repeatedly
☐	☒	The statistical test(s) used AND whether they are one- or two-sided<br>*Only common tests should be described solely by name; describe more complex techniques in the Methods section.*
☒	☐	A description of all covariates tested
☐	☒	A description of any assumptions or corrections, such as tests of normality and adjustment for multiple comparisons
☐	☒	A full description of the statistical parameters including central tendency (e.g. means) or other basic estimates (e.g. regression coefficient) AND variation (e.g. standard deviation) or associated estimates of uncertainty (e.g. confidence intervals)
☐	☒	For null hypothesis testing, the test statistic (e.g. *F*, *t*, *r*) with confidence intervals, effect sizes, degrees of freedom and *P* value noted<br>*Give P values as exact values whenever suitable.*
☒	☐	For Bayesian analysis, information on the choice of priors and Markov chain Monte Carlo settings
☒	☐	For hierarchical and complex designs, identification of the appropriate level for tests and full reporting of outcomes
☐	☒	Estimates of effect sizes (e.g. Cohen's *d*, Pearson's *r*), indicating how they were calculated

Our web collection on statistics for biologists contains articles on many of the points above.

Software and code

Policy information about availability of computer code

Data collection	EPC10 amplifier controlled by Pulse software with a Digi LIH1600 interface (HEKA, Lambrecht, Germany), microCT system (mCT40, SCANCO MEDICAL, Switzerland), Interactive Data Language (IDL, Research Systems)
Data analysis	BioquantOsteo Analysis System, GraphPad Prism 9.0

For manuscripts utilizing custom algorithms or software that are central to the research but not yet described in published literature, software must be made available to editors and reviewers. We strongly encourage code deposition in a community repository (e.g. GitHub). See the Nature Portfolio guidelines for submitting code & software for further information.

Data

Policy information about availability of data

All manuscripts must include a data availability statement. This statement should provide the following information, where applicable:

- Accession codes, unique identifiers, or web links for publicly available datasets
- A description of any restrictions on data availability
- For clinical datasets or third party data, please ensure that the statement adheres to our policy

All relevant data are available from the corresponding author upon reasonable request. Source data are provided in the supplementary information file. The microarray datasets that support the findings of this study have been deposited in the Gene Expression Omnibus (GEO) repository, the accession codes is GSE193800.

Field-specific reporting

Please select the one below that is the best fit for your research. If you are not sure, read the appropriate sections before making your selection.

☒ Life sciences ☐ Behavioural & social sciences ☐ Ecological, evolutionary & environmental sciences

For a reference copy of the document with all sections, see nature.com/documents/nr-reporting-summary-flat.pdf

Life sciences study design

All studies must disclose on these points even when the disclosure is negative.

Sample size	Sample sizes were indicated in the legend of each Figure and Supplementary Figure. No statistical methods were used to predetermine sample sizes. We determined sample sizes as follows: for cell culture experiments, we performed at least triplicate experiments and for animal phenotype analysis at least n=5 for each treatment group, to meet the minimal requirements for statistical analysis. Human sample size was chosen as a result of previous experience regarding data variability in similar models and experimental set-ups (Reference 51).
Data exclusions	No data were excluded
Replication	For all data presented in the manuscript, we examined at least three independent biological samples (three different mice at one time) to ensure the reproducibility. For each series of the experiments, all attempts at replication were successful.
Randomization	Animals and cells were randomly assigned to the experimental groups.
Blinding	Investigators were blinded to groups allocation during data collection.

Reporting for specific materials, systems and methods

We require information from authors about some types of materials, experimental systems and methods used in many studies. Here, indicate whether each material, system or method listed is relevant to your study. If you are not sure if a list item applies to your research, read the appropriate section before selecting a response.

Materials & experimental systems

n/a	Involved in the study	
☐	☒	Antibodies
☒	☐	Eukaryotic cell lines
☒	☐	Palaeontology and archaeology
☐	☒	Animals and other organisms
☐	☒	Human research participants
☒	☐	Clinical data
☒	☐	Dual use research of concern

Methods

n/a	Involved in the study	
☒	☐	ChIP-seq
☒	☐	Flow cytometry
☒	☐	MRI-based neuroimaging

Antibodies

Antibodies used	rabbit anti-Ano1 (1:1000, abclonal, CatNo. A10498, polyclonal), rabbit anti-p-Syk antibody (1:1000, abclonal, CatNo.AP0501, polyclonal),rabbit anti-Syk antibody (1:1000, abclonal, CatNo.A2123, polyclonal), rabbit anti-Clcn4 antibody (1:1000, abclonal, CatNo.A13790, polyclonal), rabbit anti-Clcn7 antibody (1:1000, abclonal, CatNo.A6886, polyclonal), rabbit anti-CFTR antibody (1:1000, abclonal, CatNo.A8386, polyclonal), rabbit anti-p-Akt antibody (1:1000, Cell Signaling Technology, CatNo.4060, polyclonal), rabbit anti-Akt antibody (1:1000, Cell Signaling Technology, CatNo.2920, polyclonal), rabbit anti-p-CaMKIV antibody (1:1000, ImmunoWay, CatNo.YP0043, polyclonal), rabbit anti-CaMKIV antibody (1:1000, Cell Signaling Technology, CatNo.4032, polyclonal), rabbit anti-Creb antibody (1:1000, Cell Signaling Technology, CatNo.9197, Monoclonal, clone NO. 48H2), rabbit anti-p-Creb antibody (1:1000, Cell Signaling Technology, CatNo.9198, Monoclonal,clone NO.87G3), rabbit anti-Plcγ2 antibody (1:1000, Cell Signaling Technology, CatNo.55512, Monoclonal, clonal NO. E5U4T), rabbit anti-p-Plcγ2 antibody (1:1000, Cell Signaling Technology, CatNo.3871, polyclonal), rabbit anti-Btk antibody (1:1000, Cell Signaling Technology, CatNo.5082, polyclonal), rabbit anti-Btk antibody (1:1000, Proteintech, CatNo.21581-1-AP, polyclonal), rabbit anti-TRAF6 antibody (1:200, Abcam, CatNo.ab137452, polyclonal),mouse anti-RANK antibody (1:200, Abcam, CatNo.ab13918, polyclonal),rabbit anti-Gapdh antibody (1:5000, Abways,CatNo.AB0036, Monoclonal).
Validation	More detailed information about these antibodies are available on these manufacturers' websites.

Animals and other organisms

Policy information about studies involving animals; ARRIVE guidelines recommended for reporting animal research

Laboratory animals	We used genetically modified mice (mus musculus) for this study. Most of the mouse lines have been backcrossed to a C57/BL6 background.
Wild animals	The study did not involve wild animals.
Field-collected samples	The study did not involve samples collected from the field.
Ethics oversight	All the experimental procedures were approved by the Committees of Animal Ethics and Experimental Safety of China Astronaut Research and Training Center.

Note that full information on the approval of the study protocol must also be provided in the manuscript.

Human research participants

Policy information about studies involving human research participants

Population characteristics	The bone tissues of 17 non-osteoporotic people and 15 osteoporotic patients with fracture at between 60 and 80 years of age.Non-osteoporotic human and osteoporotic patients who had fracture caused by falling without obvious violence were included in our study. Patients who subjected to diabetes, malignancy, hyperparathyroidism and other sever bone diseases were excluded from our study. We also excluded the patients who had taken glucocorticoids, estrogen or anti-osteoporosis drugs within 1 year.
Recruitment	All patiens in this study were from Peking University the Third Hospital, they were not in any clinical trails.
Ethics oversight	The study protocol conformed to the ethical guidelines of the 1975 Declaration of Helsinki and all the clinical procedures were approved by Peking University the Third Hospital.<br>This statement is included in the manuscript.

Note that full information on the approval of the study protocol must also be provided in the manuscript.
